# Detection of concealed knowledge via the ERP‐based technique *Brain Fingerprinting*: Real‐life and real‐crime incidents

**DOI:** 10.1111/psyp.14110

**Published:** 2022-06-07

**Authors:** M. Usman Afzali, Alex P. Seren‐Grace, Robin W. Palmer, Ewald Neumann, Sarah Makarious, Debra Wilson, Richard D. Jones

**Affiliations:** ^1^ School of Psychology, Speech and Hearing University of Canterbury Christchurch New Zealand; ^2^ School of Law University of Canterbury Christchurch New Zealand; ^3^ New Zealand Institute of Language, Brain, and Behaviour Christchurch New Zealand; ^4^ School of Electrical and Computer Engineering University of Canterbury Christchurch New Zealand; ^5^ School of Medicine University of Otago Christchurch New Zealand; ^6^ New Zealand Brain Research Institute Christchurch New Zealand

**Keywords:** Brain Fingerprinting, concealed information test, ERP, investigation, P300

## Abstract

Brain Fingerprinting (BFP) is an electroencephalogram‐based system used to detect knowledge, or absence of knowledge of a real‐life incident (e.g., a crime) in a person's memory. With the help of BFP, a potential crime suspect can be classified as possessing crime‐related information (Information‐Present), not possessing crime‐related information (Information‐Absent), or Indeterminate (BFP unable to classify a subject). In the lab setting, we compare the ground‐truth of a subject (i.e., real‐life involvement in an incident) against their classification based on BFP testing. We report two studies: *replication of BFP with university students* (Study 1) and *replication of BFP with parolees* (Study 2). In Study 1, we tested 31 subjects (24 females, seven males, mean age = 21.3) on either their own or another subject's real‐life incident. BFP correctly classified nine Information‐Present and 18 Information‐Absent subjects, but with one false positive and three exclusions. In Study 2, we tested 17 male parolees (mean age = 47.5) on their own or another parolee's crime incident. BFP correctly classified two Information‐Present and six Information‐Absent subjects. However, there was also one false positive classification and three Indeterminates. Additionally, we identified three subjects who could not complete the BFP testing and two exclusions. We posit that BFP is not yet at a stage to be considered a robust and accurate crime‐detection tool as claimed in former articles. Nevertheless, after addressing the limitations, BFP has considerable potential as an information detection tool in forensic investigations, especially for detecting idiosyncratic crime‐relevant knowledge in a perpetrator, in addition to helping to confirm the accuracy of a suspect's claim of innocence.

## Abstract 2

Despite claims of 100% accuracy by the proponents of Brain Fingerprinting, our findings include the first documented misclassifications, Indeterminates, and inabilities to carry out the Brain Fingerprinting test. Notwithstanding, all other subjects were correctly classified by the knowledge detection tool. Hence, we consider Brain Fingerprinting has many clear intrinsic strengths and we contend that, with further refinements and independent validations, it has considerable potential as a powerful new tool in forensic investigations.

## 1 INTRODUCTION

The detection of deception, as well as wrongful convictions, has been of keen interest to researchers and law enforcement agencies over several decades. Figures from the mid‐20th century until recently show that hundreds of innocent people were convicted in West Germany during the 1950s and 1960s (Nose, 1981) and the United States (The National Registry of Exonerations, 2020). Such wrongfully convicted prisoners spent an average of 9 years in United States prisons before being acquitted (Johnson, 2020). The Michigan Law Innocence Clinic (2021) lists “Junk science” (“untested or unproven theories when presented as scientific fact” [Stevenson, 2010]) as one of the underlying causes of false convictions, along with problems with eyewitness testimony, wrongful confessions, state misconduct to push for convictions despite weak evidence, prosecutorial misconduct in exercising the discretion to prosecute despite weak evidence, dishonest informants, and inadequate representation by counsel. They contend that many forensic methods have no scientific authentication and possess poor validity, which results in incorrect testimonies by forensic experts leading to false convictions.

The urgency with which these topics have been pursued has increased since September 11, 2001, and the subsequent rise in terror attacks worldwide. This has led to the development of a broad range of instruments, technologies, and techniques which have been put forward in attempts to ascertain the presence, or absence, of guilty knowledge possessed by suspected criminals.

One such instrument is the Concealed Information Test (CIT), also known as the Guilty Knowledge Test. The CIT procedure presents a subject with facts pertaining to an event or crime, and physiological and behavioral measures (e.g., elevated autonomic nervous system response levels) are used to ascertain whether the subject recognizes these facts (Lykken, 1959, 1960). The Concealed Information Test is considered a far more reliable method than the Control Question Test (i.e., the standard “polygraph”) by many in the scientific community (Ben‐Shakhar, 2012; Ben‐Shakhar & Elaad, 2003; Gamer, 2011; MacLaren, 2001; Meijer et al., 2007; Verschuere et al., 2004, 2011).

Current CIT research often incorporates event‐related brain potentials (ERPs) (e.g., Farwell, 2012; Farwell & Donchin, 1991; Funicelli et al., 2021; Rosenfeld, 2020; Rosenfeld et al., 1988), using an endogenous component of ERP known as the P300. Originally described by Sutton et al. (1965), the P300 is a positive brain potential with a maximum in the mid‐line parietal region (Pz) from 300 to 800 ms post‐stimulus, occurring when familiar significant information is presented infrequently among more frequent nonmeaningful stimuli (Donchin et al., 1986; Johnson, 1986). The items that are meaningful to the subject (e.g., autobiographical information; Berlad & Pratt, 1995; Gray et al., 2004) elicit a P300 response, but they will not elicit a similar response in those with no knowledge of the event. When these items are presented among a series of irrelevant pieces of information in a criminal event setting, details of a crime committed should elicit a P300 *only* from subjects in possession of knowledge about that crime. Early research analyzing the P300 to detect concealed knowledge found “guilty” subjects who enacted a mock‐crime later displayed P300 to crime‐relevant stimuli, whereas subjects who had not enacted the mock‐crime produced no P300 to the crime‐relevant information (Farwell & Donchin, 1991; Rosenfeld et al., 1988).

One prominent method pioneered by Farwell and colleagues (Farwell & Donchin, 1991; Farwell & Smith, 2001) is known as “Brain Fingerprinting” (BFP). BFP is a forensic brainwave analysis (FBA) system that utilizes “P300‐MERMER” ERP measures to determine whether or not someone recognizes information from a certain real‐life incident. MERMER stands for *memory and encoding related multifaceted electroencephalographic response* and is *late negative potential* (LNP) that extends up to 1200–1500 ms after presentation of the stimulus (Farwell, 2012; Farwell et al., 2013, 2014; Farwell & Smith, 2001). It is worth noting that the initial BFP research by Farwell and Donchin (1991) analyzed the well‐established P300 ERP component only (300–900 ms post‐stimulus). However, the application of BFP to specific real‐life situations often requires the use of stimuli consisting of long words, full names, or entire phrases, which take longer to read and process than shorter ERP stimuli, thus necessitating longer intervals between stimulus presentations, and a longer segment of brainwave response data recorded for each trial (Farwell, 2012; Farwell et al., 2013, 2014; Farwell & Smith, 2001). These components, along with phasic changes which appear in the structure and frequency of the signal, are grouped together as a phenomenon Farwell refers to as *P300‐MERMER* (Farwell, 2012).

BFP employs a version of the Concealed Information Test known as the *classification CIT*. Subjects are presented with three types of stimuli in BFP: *probes* (items of information that only a person intimately involved with the crime or incident in question would know (e.g., investigators, witnesses, and perpetrators), *targets* (information that the subject definitely knows, regardless of whether they were involved in the incident or crime), and *irrelevants* (information that has no relevance to the crime, and is not personally significant to the subject in any other way) (Farwell, 2012; Farwell & Donchin, 1991; Farwell & Smith, 2001). All subjects are familiarized with target stimuli, which are made significant in the context of the test by experimental instructions. Probe items are expected to elicit a P300 in subjects with concealed knowledge (Information‐Present, or IP subjects), whereas, to “innocent” (Information‐Absent, or IA) subjects, probe items will be indistinguishable from irrelevant items.

The resulting ERPs are compared using a double‐centered correlation method with 1000 iterations of bootstrapping (Allen & Iacono, 1997; Farwell et al., 2013; Farwell & Donchin, 1991; Farwell & Smith, 2001; Rosenfeld & Donchin, 2015; Wasserman & Bockenholt, 1989). This results in either Information‐Present classification (IP_C_), Information‐Absent classification (IA_C_), or Indeterminate classification. If there is at least 90% bootstrapping probability that the correlation between the average ERP responses to probes and targets is higher than the correlation between the average ERP responses to probes and irrelevants, the subject is classified as IP_C_. In other words, probe and target stimuli are registered similarly and elicit corresponding ERP responses, meaning that the subject possesses the crime/incident‐related information. If the bootstrapping probability is between 30% and 90%, BFP is considered unable to determine if a subject is IP_C_ or IA_C_, and the BFP determination is called Indeterminate. If there is a 30% or lower bootstrapping probability that the correlation between the average ERP responses to probes and targets is higher than the correlation between the average ERP responses to probes and irrelevants, the subject is classified as IA_C_. That is, there is at least a 70% bootstrapping probability that the correlation between the average ERP responses to probe and irrelevants is higher than the correlation between the average ERP responses to probes and targets. In IA_C_ classification, probe and irrelevant stimuli elicit similar ERP responses (Farwell, 2009, 2012; Farwell & Donchin, 1991; Farwell & Smith, 2001).

BFP analysis has reportedly produced no false positives and no false negatives, in differentiating knowledgeable (IP) from naïve (IA) subjects in all published studies to date (Farwell et al., 2013, 2014; Farwell & Donchin, 1991; Farwell & Smith, 2001). In an early study, 20 subjects took part in one of two fake espionage incidents, and were then tested on both incidents. There were no false positive or false negative determinations based on the ERP analyses, although five of the 40 tests resulted in Indeterminate classifications. The second study was conducted on real‐life minor crimes or transgressions (e.g., arrested due to underage drinking) of four individuals (Farwell & Donchin, 1991). Each subject was tested on their own crime incident using the BFP system and one other incident. All IP subjects were correctly determined as IP_C_, although one IA subject resulted in an Indeterminate classification. In another study, six subjects were tested on real‐life incidents using P300‐MERMER (Farwell & Smith, 2001). Three were correctly determined as IP_C_ and the other three were correctly determined as IA_C_; all with 90% or higher bootstrapping probability.

More recently, Farwell et al. (2013) conducted two studies on felony crimes of subjects conducted in a way that did not have any judicial consequences for them (study 1) and on subjects who were suspects in criminal investigations or convicted criminals who were claiming innocence, with some facing life‐imprisonment or the death penalty (study 2). The stimuli of study 2 were produced using witness and accomplice interviews, crime‐scene inspection, and the use of police and court records. In study 1, three IA and 17 IP subjects were tested, with all being correctly determined. Fourteen subjects were tested in study 2, with nine being IP and five IA. The IP subjects were offered US$100,000 if they could get the BFP system to produce a false negative result (i.e., an incorrect IA_C_) (Farwell et al., 2013). These subjects were encouraged to use countermeasures similar to those investigated by Rosenfeld et al. (2004) and Mertens and Allen (2008) such as covertly wiggling the big toe in the right or left shoe, or imagining being slapped in the face by the experimenter. Regardless of these countermeasures, all subjects were correctly classified according to their ground‐truth. Countermeasures were found to be ineffective, despite the large incentive to foil the system.

Despite being publicized as a highly accurate forensic brainwave analysis technology by Farwell, BFP has not yet gained wide acceptance as a forensic tool. Notwithstanding, Farwell (2009) reported use of the BFP in the Iowa District Court in which BFP showed that the convicted criminal Terry Harrington was IA_C_ for the crime incident and IP_C_ for his alibi incident, *Harrington* vs. *State*, 659 N.W.2d 509 (Iowa, 2003, as cited in Farwell & Makeig, 2019). Nevertheless, the Court upheld the conviction and did not change the ruling based on BFP. It was the Appeal Court that later acquitted Harrington based on evidence other than Farwell's findings. In addition, James B. Grinder pled guilty to rape and murder after BFP testing implicated him as the perpetrator (Farwell, 2012; Farwell et al., 2013; Moenssens, 2001). Brain Fingerprinting analysis can focus solely on the P300 element (300–900 ms following stimulus presentation), but Farwell and colleagues assert that analyzing the full P300‐MERMER epoch (300–1800 ms post‐stimulus) gives higher accuracy, higher bootstrapping probability, and lower incidence of Indeterminate classifications in ERP‐based knowledge detection (Farwell, 2012; Farwell et al., 2013). Although some researchers debate the value of the P300‐MERMER (Rosenfeld, 2005), Farwell's claims appear to be supported by the fact that previous BFP studies taking the entire MERMER into account have produced zero determination errors and zero Indeterminates (Farwell et al., 2013, 2014; Farwell & Smith, 2001), whereas early studies focusing solely on the P300, while still reporting 100% accuracy, resulted in a small proportion of Indeterminates (Farwell & Donchin, 1991). Recent field studies comparing the two types of analysis found that both the P300 alone and P300‐MERMER analyses were 100% accurate in their determinations (Farwell et al., 2013).

Brain Fingerprinting has been the subject of robust debate in the scientific community (Farwell, 2011; Farwell et al., 2014; Farwell & Richardson, 2013; Meijer et al., 2013; Rosenfeld, 2005; Rosenfeld et al., 2004, 2008). Many criticisms center around a lack of independent review or validation of the Brain Fingerprinting technique currently in the literature (e.g., Harrington vs. State, Iowa, 2003, as cited in Farwell & Makeig, 2019; Rosenfeld, 2005; Satel & Lilienfeld, 2013), and argue that claims made by Farwell and colleagues overstate its accuracy (Meijer et al., 2013; Rosenfeld, 2005; Rosenfeld et al., 2004, 2008; Satel & Lilienfeld, 2013). Some researchers, endeavoring to replicate BFP protocols, have found much lower accuracy with their methods (e.g., Bergström et al., 2013; Rosenfeld et al., 2004, 2007) than reported by BFP researchers. In addition, further studies (Bergström et al., 2013; Funicelli et al., 2021; klein Selle et al., 2021; Rosenfeld et al., 2004) determined that P300‐based information detection techniques, such as BFP and Complex Trial Protocol (another P300‐based forensic brainwave analysis system used by Rosenfeld et al., 2004) could be susceptible to behavioral as well as cognitive countermeasures and to misleading information. However, proponents of BFP have dismissed these findings on the basis that the procedures and data analysis used in these studies deviated substantially from those used in BFP research (Farwell et al., 2013; Farwell & Richardson, 2013). Farwell et al. (2013) set out 20 scientific standards (20SS hereafter—see Appendix A) which stipulate necessary guidelines to be followed in order for any replication to be an accurate interpretation of Brain Fingerprinting protocols, asserting that any protocol which meaningfully deviates from these standards does not constitute ‘Brain Fingerprinting’. Since these standards were not followed by Bergström et al. (2013), their findings cannot be generalized to BFP according to Farwell et al. (2013).

Alongside this controversy, it is crucial to note that nearly all published articles on BFP to date have not been independent of Farwell (the inventor and main stakeholder of BFP). The only exception is Allen and Iacono (1997) that applied BFP's mathematical algorithm to data from one of their own studies (Allen et al., 1992) to compare the accuracy of BFP's algorithm to that of their Bayesian‐based algorithm. They reported a very high classification accuracy for both the BFP algorithm and for their own algorithm. On the other hand, the Complex Trial Protocol has been independently tested on multiple occasions (Funicelli et al., 2021; Lukács et al., 2016). Moreover, the majority of previous BFP studies have not recruited convicted criminals as their study subjects except for the testing of some suspects and criminals by Farwell et al. (2013). The nature of the examined crimes and the exact number of subjects in each sub‐condition (being a suspect, a convict claiming innocence, facing life‐imprisonment, or death penalty) was, however, not reported by (Farwell et al., 2013).

The present project aimed to address major points of controversy around BFP in two ways. First, we wished to provide an independent assessment of the efficacy of BFP testing protocols, as none of the research team members have any association with Farwell apart from their formal training to ensure close adherence to the BFP scientific standards. Second, we wished to replicate the BFP testing protocol on university students (Study 1) as well as convicted criminals (Study 2). In Study 1, we tested students on their own or other students' life incidents. In Study 2, we tested criminals on their own or other criminals' confessed crime incidents. It was deemed appropriate to recruit criminals who had already faced the consequences of their crimes, so that the findings could not affect them in any way. If BFP is as reliable and accurate as claimed by Farwell and colleagues, BFP testing would accurately classify all IP subjects as Information‐Present (IP_C_) and all IA subjects as Information‐Absent (IA_C_), with no false positives, and no false negatives in both Studies 1 and 2.

## 2 METHOD

### 2.1 Participants

Study 1 participants were 31 University of Canterbury students, aged between 18 and 29 years (*M* = 21.3), comprising 24 females and seven males. Study 2 had 17 male adult ex‐prisoners from the Salisbury Street Foundation half‐way house in Christchurch, New Zealand. They were aged 27–75 years (*M* = 47.5) and were all convicted criminals on parole from Christchurch Men's Prison for separate serious crimes including homicide, robbery, arson, assault, and sexual offenses. No exclusion criteria were applied for either study, although Study 2 subjects were required to have a minimum standard of reading comprehension, and be confined to, residents of, or had linkage to the half‐way house. A University of Canterbury staff member with connections to the half‐way house was appointed to help with identifying, recruiting, and escorting the subjects of Study 2.

All subjects volunteered to participate. They were given an information sheet several days prior to testing, and those who agreed to take part in this study signed consent to participate in the study. Study 1 subjects received a NZ$60 voucher and Study 2 subjects received a NZ$100 voucher as gratuity. Both studies were approved by the Human Ethics Committee of the University of Canterbury (HEC 2019/63 and HEC 2019/152).

### 2.2 Material and apparatus

The BFP hardware and software, for data acquisition and analysis, were leased from Brain Fingerprinting, LLC (Seattle). Separate Cognionics (San Diego) software was used to measure electrode impedances on the Cognionics EEG‐headset.

The tester ran the experiment from a screen on a Windows PC and the subject sat 60 cm in front of a separate screen, on which all experimental instructions and stimuli were presented. The ERP data were collected using a wireless, custom‐made, dry‐electrode EEG headset that recorded mid‐line scalp locations (frontal = Fz, central = Cz, and parietal = Pz, International 10–20 System). Electrooculogram (EOG) signals were collected from Fp1 and Fp2 to detect eye‐blink artifacts. Linked mastoid electrodes were used as the signal reference. The right‐ and left‐hand buttons on an Xbox controller were used to obtain behavioral responses.

### 2.3 Design

The ground‐truth status (IP vs. IA) was the between‐subjects independent variable and the stimulus type (probe vs. target and irrelevant) was the within‐subjects independent variable. The ERP response leading to a BFP classification (IP_C_, IA_C_, and Indeterminate) was the dependent variable.

Study 1 subjects were divided into three groups. One group was assigned to each BFP tester (four subjects in Test Group A, 16 in Test Group B, and 10 in Test Group C). Uneven numbers were a product of tester availability. Subjects were interviewed about a memorable event involving themselves and no other subject in their test group. Nine of these events were chosen at random and a BFP test formed for each selected incident (one incident for Test Group A, five incidents for Test Group B, and three for Test Group C).

Study 2 subjects were also divided into three groups. One group was assigned to each BFP tester (six subjects in Test Group A, six in Test Group B, and five in Test Group C). All subjects were interviewed on one of their confessed crimes, selected using their criminal records that involved no other subject within their test group. The stories told by these subjects were corroborated with their corresponding police files to ensure accuracy. Initially, three of these incidents, *Flatmate Assault*, *Revenge*, and *Robbery*, were randomly chosen and a BFP test was formed for each of them. Later, two more tests were added: The *Armor Guard Heist* incident replaced *Revenge*. The reason being that the IP subject of the *Revenge* incident failed to attend the experiment. Since other subjects of this incident had not been tested at that stage, we replaced it with the *Armor Guard Heist* incident. The second added test was *Stolen Dog* incident. It was used to re‐test one of the subjects (details in Results).

To ensure tester blindness, subjects were interviewed and the BFP test stimuli were formulated for each incident only by testers who would not go on to administer the BFP test to the subject in question, and by the study coordinator. For each incident, one subject with the knowledge of the event in question (IP) was tested, and either two or three (Study 1) or four or five (Study 2) subjects with no knowledge of the event (IA) were tested. The sole role of the coordinator was to oversee the study, recruit study subjects, oversee stimuli development, and most importantly, ensure that the 20SS were adhered to by the testers. The BFP testers and the study coordinator had been trained and certified by Dr Farwell and followed the BFP testing manual and 20SS to ensure consistency and robustness of the testing procedure.

### 2.4 Stimuli

Subjects were interviewed by two trained BFP testers, and the project coordinator. To ensure tester blindness, the specific BFP tester assigned to test any given subject was not permitted to be present during that participant's interview. During the interview session, participants were prompted to recount a “memorable event” from their lives with what, when, where, who, and how questions. Subjects recounted a wide variety of different events. Some of these events involved criminal wrongdoing, while others did not. Subjects were assured that all recounted events would be anonymized and that they would face no legal consequences for criminal activity disclosed to interviewers. They were also advised that, if they chose to disclose an event involving serious criminal behavior, they should choose a crime for which legal repercussions had already been faced, to avoid a conflict of interest for interviewers in keeping their stories confidential. Events that were randomly selected for incorporation into BFP tests included situations where subjects were involved in a serious car accident, or other near‐death experience, witnessed an alarming incident of a building engulfed in flames, watched the disappearance of a beloved pet sucked into a whirlpool, encountered a man passing away from a heart attack, and being arrested for assault and property destruction due to a drug‐induced psychosis.

20SS (Farwell et al., 2013) comprise Farwell's specifications on ensuring effective BFP stimuli formation. BFP tests comprised six probes, six targets, and 24 irrelevants, consistent with the recommended minimum number of each (3, 3, and 6, respectively).

For each incident in both studies, a set of BFP stimuli was formed. Probe and target items were selected from information gathered during the BFP interviews. Selected probe items were pieces of information that would only be recognized as significant to the incident in question by someone who had intimate knowledge of the incident (i.e., was present at the time). Target items were also significant pieces of information about the incident in question, and all subjects were familiarized with these targets during the administration of BFP testing instructions, so that targets ultimately would be recognized as significant to the incident under investigation by all subjects. Irrelevant items were inconsequential pieces of information that had no relevance to the incident under investigation but were designed to be indistinguishable from probes to any person without intimate knowledge of the incident (see Tables 1 and 2 for examples from both studies).

**TABLE 1:** Example of stimuli for study 1

Probe/Target	Original stimulus	Description	Irrelevant 1	Irrelevant 2
Probe	Name^a^	A friend who was present during the incident	Sapphire McCarthy	Jenelle Fitzpatrick
Target	Name^a^	A friend who was present during the incident	Jazlyn Ryan	Clarissa Wheeler
Probe	Name^a^	A person who was present during the incident	Leo Sutton	Jackson Newman
Target	Name^a^	Stepfather who was called on the phone after the incident	Kaine	Glen
Probe	Name^a^	A parent who helped retrieve the vehicle	Troy	Saul
Target	Name^a^	A person who helped retrieve the vehicle	Victor Spears	Gavin Barr
Probe	Rakaia River	Where the group spent time before the incident	Waipara valley	Papanui shops
Target	Isuzu MU	The type of vehicle involved	Subaru Legacy	Land Cruiser
Probe	Inexperienced driver	A factor which contributed to the incident happening	Brake failure	Careless pedestrian
Target	Swimming	An activity the group did before the incident	Climbing	Shopping
Probe	Fence	Something the vehicle collided with	Sheep	Barn
Target	Southbridge	Where the group traveled from	Lincoln	Amberley

^a^ Six names of real people provided by subjects have been redacted to ensure confidentiality.

**TABLE 2:** Example of stimuli for study 2

Probe/Target	Original stimulus	Description	Irrelevant 1	Irrelevant 2
Probe	Name^a^	Name of the subject's sister	Marlene Gonzales	Tanya Lacrosse
Target	England Street	Road in which the subject lived at the time	Jackson Drive	Hillside Avenue
Probe	Name^a^	Name of the subject's girlfriend	Tilly Wilson	Mary Elwood
Target	Petrol Station	The type of place the subject planned to rob	Supermarket	Estate Agency
Probe	Army	The organization that offered the subject's sister a job	Navy	Air Force
Target	Park	The place where the robbery incident happened	Café	Bank
Probe	Kitchen knife	The weapon used by the subject's sister	Claw hammer	Pepper spray
Target	Handbag	The main item stolen in the robbery incident	Briefcase	Money box
Probe	Hit with fists	How the subject assaulted a robbery victim	Shot with gun	Struck with bat
Target	Vomited	A physical reaction the subject's sister had after the robbery	Fainted	Cried
Probe	Wallet	The stolen item the subject put in his pocket	Wristwatch	Necklace
Target	Marian College	The school grounds through which the subject ran after robbery	Buxton High	Stanton Primary

^a^ Two names of real people provided by subjects have been redacted to ensure confidentiality.

All subjects, whether IA or IP, were expected to recognize targets as significant. We also expected that IA subjects would not recognize probes or irrelevants as significant, and that their ERP responses to probes would more closely resemble ERP responses to irrelevants (i.e., no P300‐MERMER) than responses to targets, resulting in a BFP determination of Information‐Absent (IA_C_).

IP subjects were expected to recognize targets *and probes* as significant, due to their familiarity with the incident under investigation. If Farwell's findings generalized to the present paradigm, it was expected that IP subject responses to probes would more strongly correlate with their responses to targets (i.e., P300‐MERMER) than responses to irrelevants, resulting in a BFP determination of Information‐Present (IP_C_).

The incidents used in BFP testing tend to be quite idiosyncratic and subjective to subjects' experiences. That is, an incident being tested for one group of participants (one IP and several IAs) in a study will usually be very different from another incident in the same study. This could create questions of inconsistency between incidents from a scientific methodological point of view. Because we were testing multiple incidents in each study, we could not test all subjects on one incident. However, we did ensure that the stimuli were developed in a systematic and objective manner. These following measures were taken to ensure this consistency:

1. The stimuli were developed by two testers and were peer‐reviewed by the study coordinator to ensure consistency and objectivity.
2. The irrelevant items for the human names were formulated based on a database that lists names and surnames in terms of popularity. For instance, if a name narrated by a subject was Martin Jackson, the database shows popularity ranks of 190 and 13, respectively. A suitable irrelevant for this would be Johnny Anderson—ranked 184 and 11, respectively, with a similar number of syllables.
3. We ensured a similar consistency for names of vehicles and places.

### 2.5 Procedure

The interviews and BFP testing were carried out on the campus of the University of Canterbury in Christchurch. Study 1 subjects made their way to the testing location themselves. Study 2 subjects in residence at the half‐way house were escorted to the campus by the half‐way house staff members, and subjects based in the community made their own way to the University for testing by appointment. The testing took place at a designated testing space at the University.

The BFP tests were carried out within 5 to 30 days following subject interviews and the structure and content of the BFP tests complied with the guidelines stipulated in Farwell's 20SS. Key elements of this testing design included:

1. Each BFP test comprised two stimulus sets: Set 1 and Set 2.
2. Each BFP test contained six unique probes, six unique targets, and 24 unique irrelevants. Of these, three probes, three targets, and 12 irrelevants were assigned to each of stimulus Sets 1 and 2 (see Tables 1 and 2 for examples).
3. Each unique probe, target, and irrelevant stimulus was presented a minimum of 20 times for each BFP test. This amounted to a minimum of 720 trials, comprising 120 probe trials, 120 target trials, and 480 irrelevant trials. Additional trials were automatically added by the BFP software to compensate for any trials rejected due to artifacts (usually due to eye‐blinking or extraneous movement by the subject).
4. Each BFP test comprised 10 blocks of trials in Study 1 and 16 blocks in Study 2, each block containing a minimum of 72 trials. All blocks presented stimulus Sets 1 and 2 in an alternating sequence. Trial blocks displayed each of the unique probes, targets, and irrelevants in the relevant stimulus set a minimum of four times.

Prior to administration of the test, each subject was met by one of the testers who had previously interviewed them. IA subjects were shown a list of the targets which would later appear on‐screen in their BFP test and were instructed to familiarize themselves with the target items. IP subjects were shown a list of the target and probe items, which they already knew. Subjects were instructed to review all items on the list they were shown, and confirm that they would recognize these items later. This *information confirmation* procedure was added to counteract the possibility of any subject lying, embellishing the truth, or guessing when questioned about details they did not honestly remember from the event recounted in their interview. Following this, they also participated in a practice BFP test without ERP data being collected to familiarize them with the experimental procedure. They were instructed to recognize the target stimuli and press the left‐hand button on the Xbox controller; and to press the right‐hand button for any other stimuli (either probe or irrelevant).

In a field setting, investigators would be able to independently verify the details of an event or crime, using police evidence, independent witness testimony, or other methods. These methods were not viable in the context of this study, so the information confirmation procedure was employed to ensure that, even if a subject had falsely recounted any detail of the event under investigation, the details which were incorporated into the BFP stimuli would be familiar to them, and significant within the context of the BFP test.

#### 2.5.1 Brain Fingerprinting test

Experimental instructions ensured that all subjects were familiarized with target items and understood their significance in the context of the incident under investigation. Subjects were informed that they would be required to recognize target items during the test. Subjects held an Xbox controller and were instructed to press a left‐hand button in response to all target items once the test commenced. The significance of the probes in the context of the incident under investigation was also explained. Probe item descriptions were shown, followed by the probe and corresponding two irrelevants, in a random order (e.g., “The support service that arrived first: NZ Police, Community Patrol, Fire Brigade”). As probe items were not identified to subjects, only subjects with prior knowledge of the event would understand which of the three items displayed was relevant. All irrelevant items that would appear during the BFP test were briefly shown in a list, and subjects were instructed to identify if any of the items were significant to them for a reason unrelated to the incident under investigation. Any items identified as personally significant to the subject for an unrelated reason were removed and replaced with an alternate irrelevant item. Subjects were instructed to read and respond to each stimulus during the BFP test with a button press, using the left‐hand button on the response controller to target items (described in experimental instructions as “items relevant to the situation under investigation”), and the right‐hand button to all other stimuli. The behavioral response task (button pushing) had no bearing on ERP recording or BFP analysis but was implemented to ensure that subjects were paying attention to, and understood the stimuli displayed on‐screen. BFP testing commenced once all experimental instructions were administered and understood by the subject.

Each block of the BFP test lasted 3–5 min and comprised targets, probes, and irrelevants, presented one at a time, randomly ordered, in white font at the center of a blue computer screen. Blocks contained a minimum of 72 trials, 1/6 of which were target trials, 1/6 probe trials, and 4/6 irrelevant trials. One stimulus set was displayed per block; stimulus Sets 1 and 2 alternated between blocks (i.e., Block 1 = Set 1, Block 2 = Set 2, Block 3 = Set 1, Block 4 = Set 2, Block 5 = Set 1 … etc.). Prior to each block, the relevance of the target items, and potential relevance of probe items (which should only be recognizable to IP subjects) in the upcoming block was signaled by the following instructions viewed and read aloud by the subjects: “Here are the items you will see in this test that are related to the investigated situation. Push the left‐hand button for the items that were on the short list of things you know about the situation, and the right‐hand button for anything else”. At this stage, a list of three item descriptions for targets and three item descriptions for probes was presented to the subjects. The *short list* in the instructions refers to the list of targets introduced to the subjects during the initial instructions. These descriptions were not accompanied by corresponding target, probe, or irrelevant items. For example, “In this test you will see: The part of the car that was damaged in the incident, The road on which the incident occurred, The model of the car involved in the incident …”, etc.

Each stimulus was preceded by a fixation cross (X) displayed in the center of the screen for 1000 ms, followed by the stimulus (target, irrelevant, or probe item) displayed for 300 ms, followed by a blank screen for 1700 ms preceding the next fixation cross (signaling the start of a new trial). Subjects were instructed to sit still and quietly, and to blink only at the appearance of the “X”, keeping their eyes open at all other times as much as possible during each block.

Any trial disrupted by extraneous artifact due to eye movement or muscle activity (amplitude >400 μV in the Fp1 channel) was rejected. Additional trials were added until artifact‐free trials in the block totaled at least 12 probe trials, 12 target trials, and 48 irrelevant trials, at which point the block was complete. In total, over the 10 blocks in Study 1 and the 16 blocks in Study 2, a minimum of 120 target trials, 120 probe trials, and 480 irrelevant trials were collected. Data were digitized at 100 Hz. Electrode‐scalp impedances were confirmed less than 10 kΩ at the beginning of testing and were rechecked during the test if necessary. Data were stored on disk for offline analysis.

#### 2.5.2 Data analysis

EEG data from the mid‐line parietal (Pz) electrode, and the EOG signals, were amplified, analog low‐pass filtered at 30 Hz, digitally low‐pass filtered at 6 Hz (3 dB cutoff), and trials with an EOG range greater than 200 μV and/or an EEG range greater than 150 μV were excluded from analysis. The analysis epoch was defined as 300–1500 ms from stimulus onset.

The BFP software does not provide information on baseline correction, eye‐movement correction, correction for amplifier drift, flatlining, etc., nor pre‐stimulus activity. The analysis report produces an *html* file that describes the number of blocks, data on behavioral accuracy, and BFP determination, and displays an ERP graph. As this project was an independent, yet direct, replication of Farwell's BFP technology, we deemed it inappropriate to analyze and report aspects that could not be obtained from the BFP software.

Analysis aimed to ascertain whether ERP responses to probes correlated more with target responses (large P300‐MERMER amplitude) or irrelevant responses (lacking a large P300‐MERMER). A bootstrapping procedure (Farwell et al., 2013, 2014; Farwell & Donchin, 1991; Wasserman & Bockenholt, 1989) and double‐centered correlation were employed to determine whether probe ERP responses were more similar to target responses (classification: IP_C_) or irrelevant ERP responses (classification: IA_C_) and compute a bootstrap probability for this classification. For each subject's data set, P probe responses, T target responses, and I irrelevants were randomly sub‐sampled, where P equals the number of probe trials in the data set, T equals the number of target trials in the data set, and I equals the number of irrelevant trials in the data set. The time‐series correlation between the response curves for probes and targets was compared to the correlation between the response curves for probes and irrelevants. This procedure was repeated 1000 times for each subject, and the number of times the probe‐target correlation was greater than the probe‐irrelevant correlation was converted to a percentage. This percentage (Prob) was interpreted as the bootstrap probability that the subject possessed concealed knowledge about the event they were tested on (Information‐Present). (100% – Prob) was interpreted as the bootstrap probability that the subject did not possess concealed knowledge about the event under investigation (Information‐Absent) (Farwell et al., 2013, 2014; Farwell & Donchin, 1991). An a priori cutoff of 90% was set for an Information‐Present classification, and a cutoff of 70% in the other direction (i.e., 100%—Pros was set for a classification of Information‐ Absent. For example, a subject with Prob = 97% would be classified as Information‐Present (IP_C_) with a bootstrap probability of 97%. A subject with Prob = 14% would be classified as Information‐Absent (IA_C_) with a bootstrap probability of 86%. Subjects falling outside of either cutoff criterion would be classified as Indeterminate (Farwell & Donchin, 1991). The BFP software, unfortunately, does not provide any more descriptive data on the nature of the bootstrapping procedure except that mentioned in Farwell's published articles (described above).

The overt behavioral aspect of data acquisition (i.e., pressing a button on the Xbox controller) ensured that subjects were paying attention to, and understood, the stimuli by pressing the appropriate button. We refer to this as *behavioral accuracy* and the frequency of correct behavioral response (i.e., pressing the left button for targets and the right button for probes and irrelevants) is converted to a percentage score that the BFP software calculates for each block, and also as an average of all blocks for a subject. This behavioral accuracy is implicit in the 20SS, but has not been reported in published BFP articles. We set an a priori criterion of ≥80% behavioral accuracy for each block for target and irrelevant stimuli, considering that sometimes a wrong button might be pressed mistakenly. We rejected blocks if behavioral accuracy was less than 80% and excluded subjects if their overall behavioral accuracy was below 80%. However, the 80% rule was not imposed on probes because although IA subjects would press the correct button (the right‐hand button) for probes, it is possible that an IP subject may confuse probes with targets due to their own prior knowledge (because of participation in the incident) and press the left‐hand button. To prevent tester bias, these accuracies were explored by the project coordinator after the testing was completed, so that the probe accuracy would not inform the tester about the ground‐truth status of a subject. If any blocks were supposed to be rejected at this stage, then new blocks would be recorded under the coordinator's direct supervision to prevent tester bias.

## 3 RESULTS

In both studies, some subjects were excluded for various reasons as follows.

### 3.1 Exclusions

Three subjects never started the test. Of these, two were scheduled for Study 1 (L08 and L12) and one for Study 2 (C17). Two other subjects (L04 in Study 1 and C04 in Study 2) had started the experiment, but did not continue to the end, which prevented recording a sufficient number of trials (100 trials required for each stimulus type, probe, target, and irrelevant according to 20SS). Thus, these two incomplete data sets could not be analyzed and were excluded.

Three others in Study 2 withdrew due to uncontrollable excessive eye‐blinking leading to eye‐fatigue at the time of testing (C05, C06, and C12). C12 was the IP subject of the *Robbery* incident and the other two were IAs of the *Flatmate Assault* incident. Excessive eye‐blinking resulted in large numbers of trials being rejected and blocks taking more than 10 min, resulting in eye‐fatigue just after 2–3 blocks.

The remaining subjects (*n* = 28 in Study 1 and *n* = 12 in Study 2) satisfactorily completed the BFP testing and their BFP findings are detailed below after a brief discussion of their behavioral accuracy.

### 3.2 BFP findings

Individual data for all subjects were analyzed utilizing the classification CIT bootstrapping procedure.

#### 3.2.1 Study 1

**FIGURE 1:**
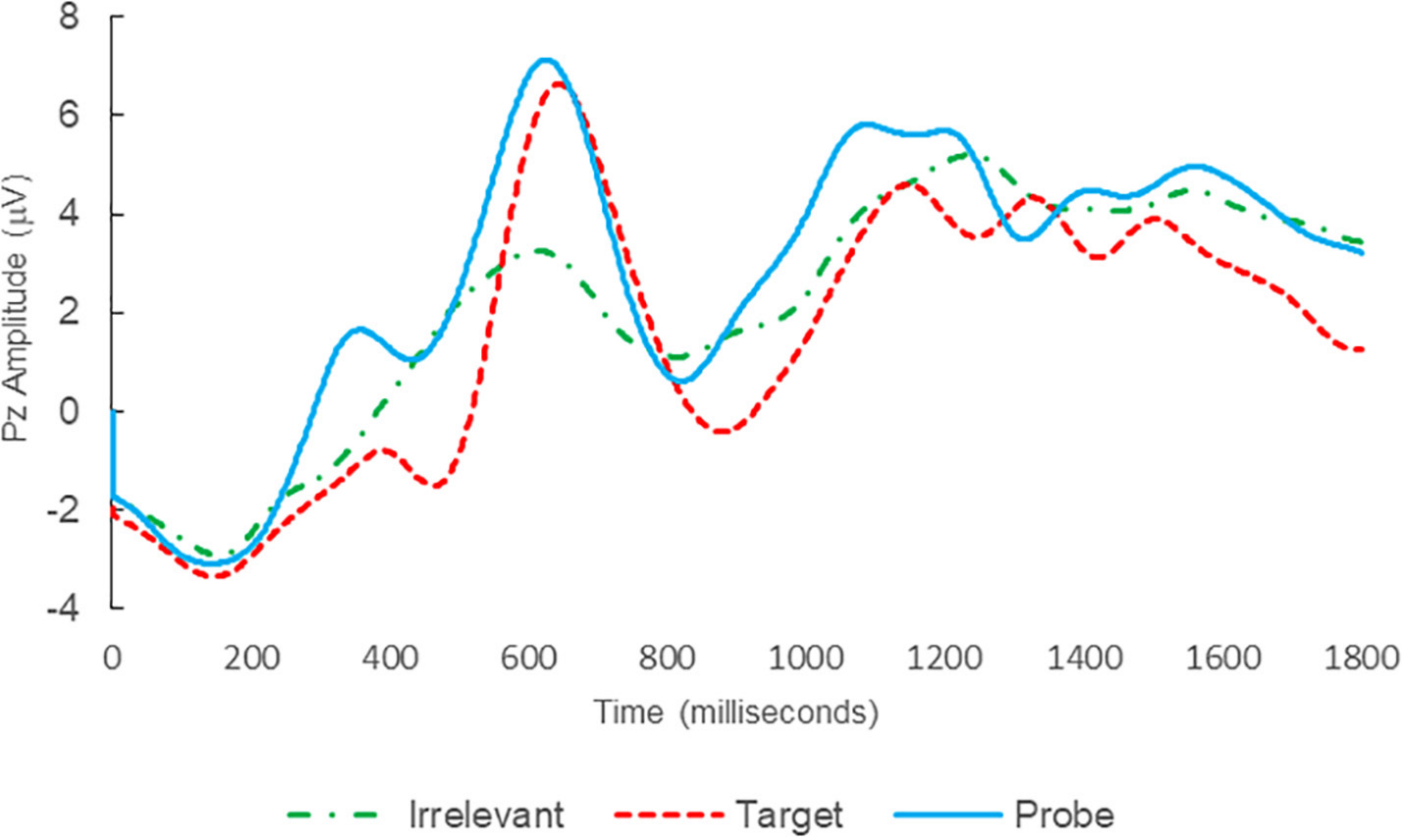
BFP response waveforms of L22 in “LSD Rampage” (IP → IP_C_).

**FIGURE 2:**
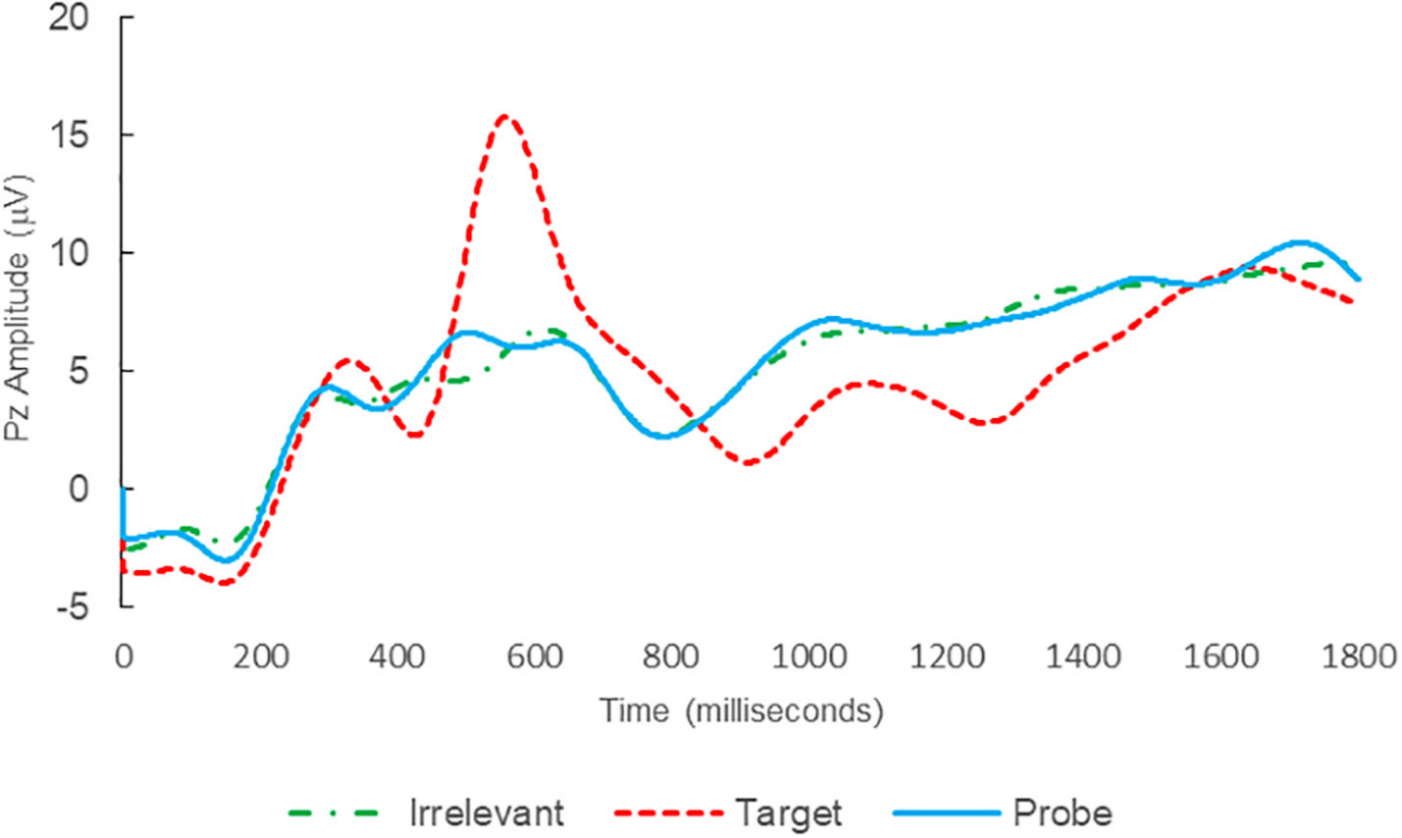
BFP response waveforms of L24 in “LSD Rampage” (IA → IA_C_).

**FIGURE 3:**
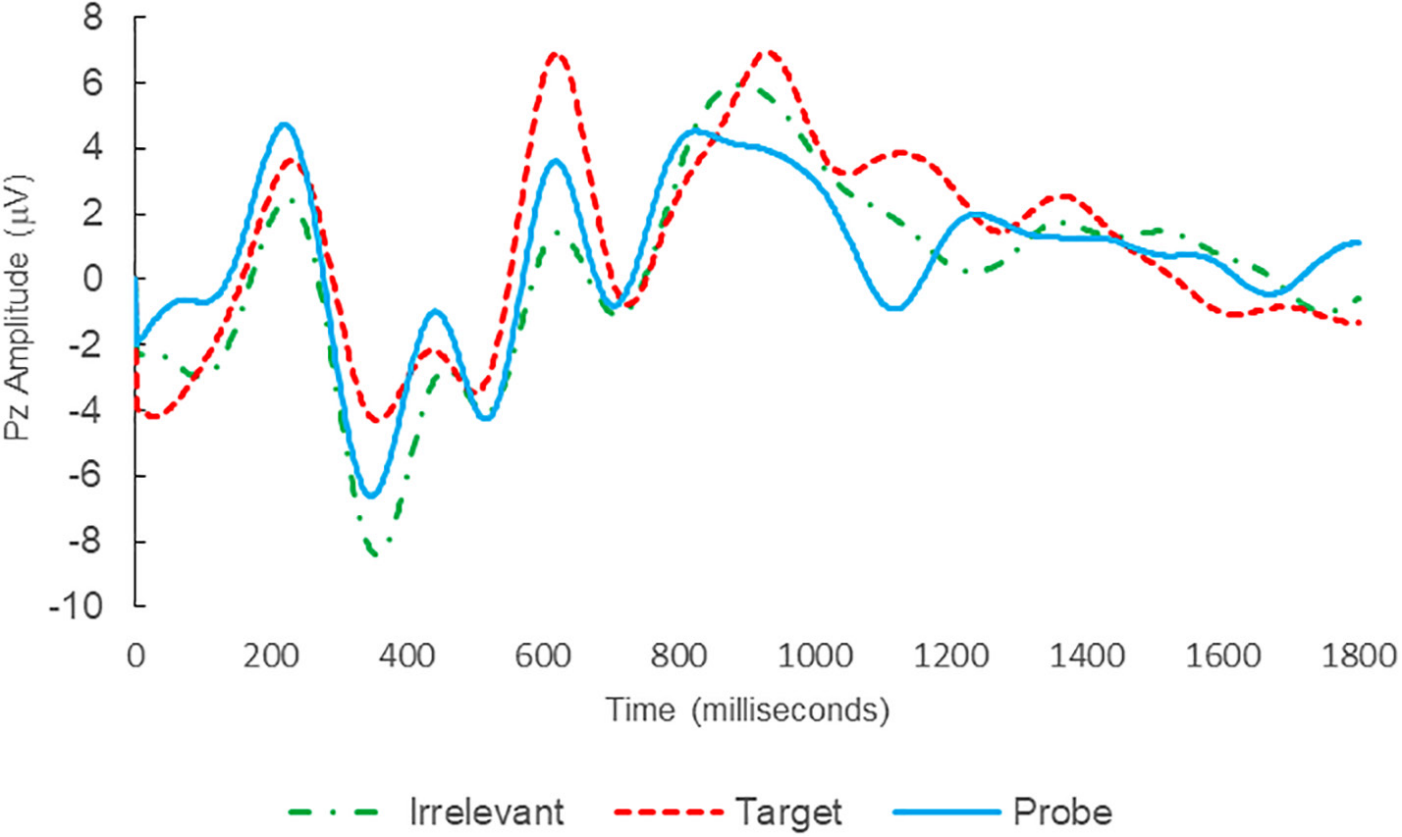
BFP response waveforms of L28 in “Dog Whirlpool” (IA → IP_C_).

Of the 28 remaining subjects in Study 1, all nine IP were correctly classified as IP_C_, with a mean bootstrap probability of 99.9%, and 18 IA subjects were correctly classified as IA_C_, with a mean bootstrap probability (correct IA_C_ classifications) of 98.2% (Table 3 and Figures 1 and 2 for example ERPs). However, one IA subject (L28) was misclassified as IP_C_, with a bootstrapping probability of 95.3% (Table 3 and Figure 3).

**TABLE 3:** Summary of Brain Fingerprinting results for study 1

Incident	Subject ID	Ground‐truth	BFP determination	Bootstrapping probability (%)
Eiffel Tower	L01	IP	IP_C_	99.9
	L02	IA	IA_C_	92.6
	L03	IA	IA_C_	99.9
	L04	IA	Excluded (<100 trials)	
Skidding	L05	IP	IP_C_	99.9
	L06	IA	IA_C_	95.9
	L07	IA	IA_C_	99.7
	L08	IA	Never participated	
Casino	L09	IP	IP_C_	99.9
	L10	IA	IA_C_	95.9
	L11	IA	IA_C_	99.7
	L12	IA	Never participated	
Red Convertible	L13	IP	IP_C_	99.9
	L14	IA	IA_C_	99.9
	L15	IA	IA_C_	99.9
Playing Pool	L16	IP	IP_C_	99.9
	L17	IA	IA_C_	99.9
	L18	IA	IA_C_	99.1
Drag Queen	L19	IP	IP_C_	99.9
	L20	IA	IA_C_	99.1
	L21	IA	IA_C_	99.9
LSD Rampage	L22	IP	IP_C_	99.9
	L23	IA	IA_C_	94.2
	L24	IA	IA_C_	96.0
Dog Whirlpool	L25	IP	IP_C_	99.9
	L26	IA	IA_C_	99.9
	L27	IA	IA_C_	98.3
	L28	IA	IP_C_ ^a^	95.3
Four Wheel Driving	L29	IP	IP_C_	99.7
	L30	IA	IA_C_	98.2
	L31	IA	IA_C_	99.9

*Note*: The letter “L” preceding each subject number refers to “Real‐Life” incidents.

Abbreviations: BFP, Brain Fingerprinting; IA, ground‐truth Information‐Absent; IA_C_, classified as Information‐Absent by Brain Fingerprinting; IP, ground‐truth Information‐Present; IP_C_, classified as Information‐Present by Brain Fingerprinting.

^a^ Red colored font shows a false positive classification.

Thus, 27 out of 28 classifications were correct (accuracy of 96.4%), and consistent with ground‐truth (i.e., whether the subject possessed concealed knowledge about the incident they were tested on, or not), with one false positive, zero false negatives, zero Indeterminates, and a mean bootstrap probability for correct determinations of 98.8%.

#### 3.2.2 Study 2

**FIGURE 4:**
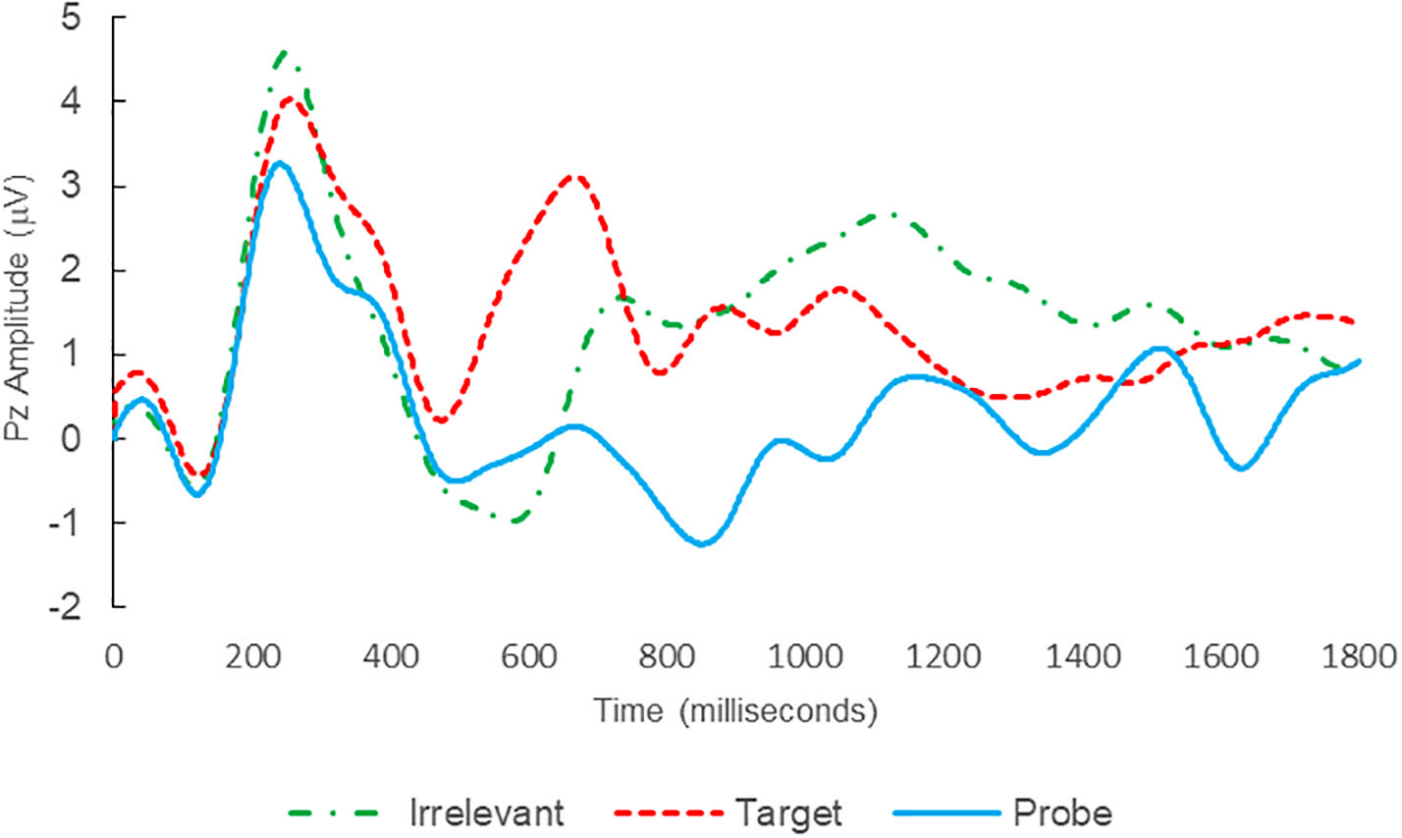
BFP response waveforms of C01 in “Flatmate Assault” (IP → IP_C_).

**FIGURE 5:**
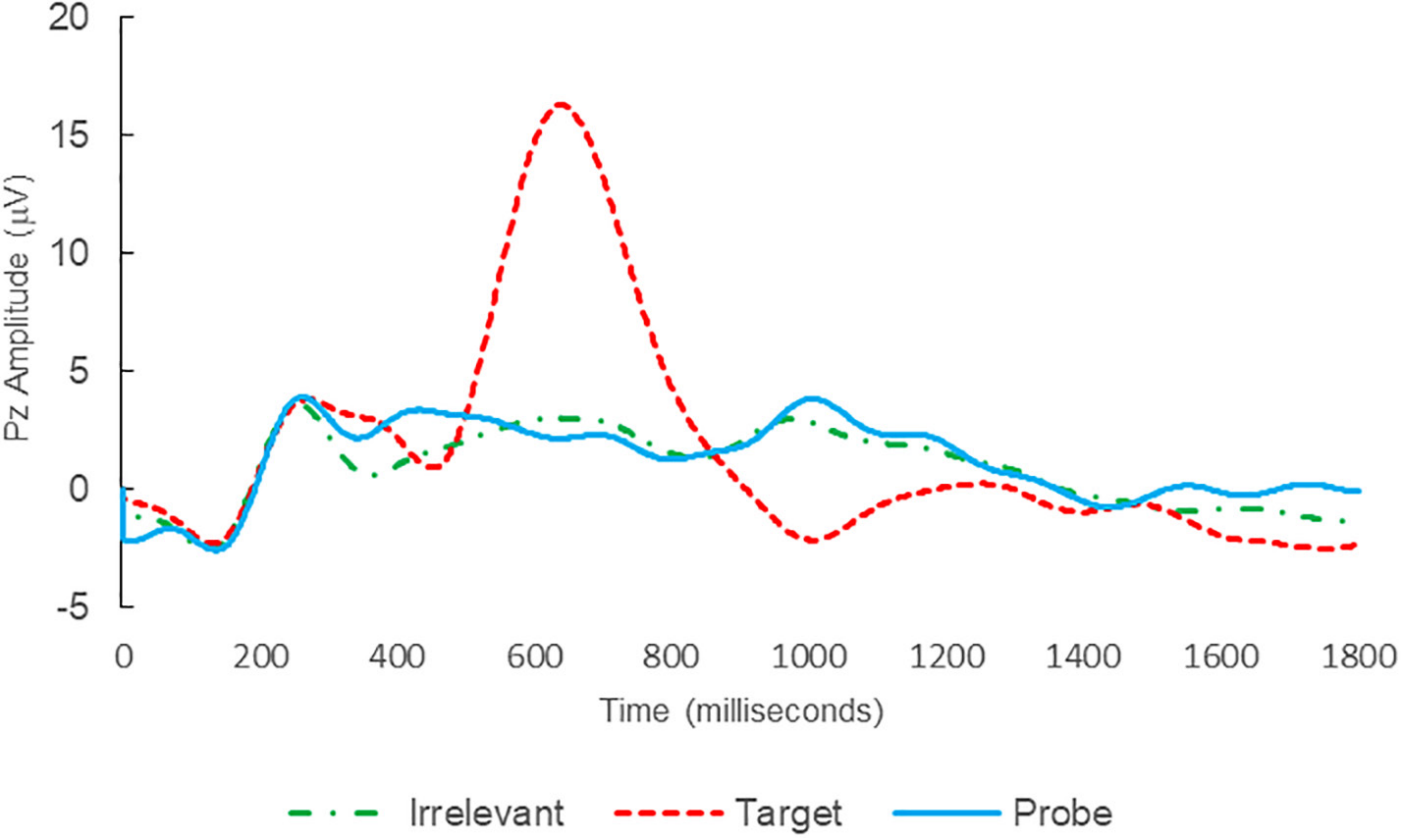
BFP response waveforms of C02 in “Flatmate Assault” (IA →IA_C_).

**FIGURE 6:**
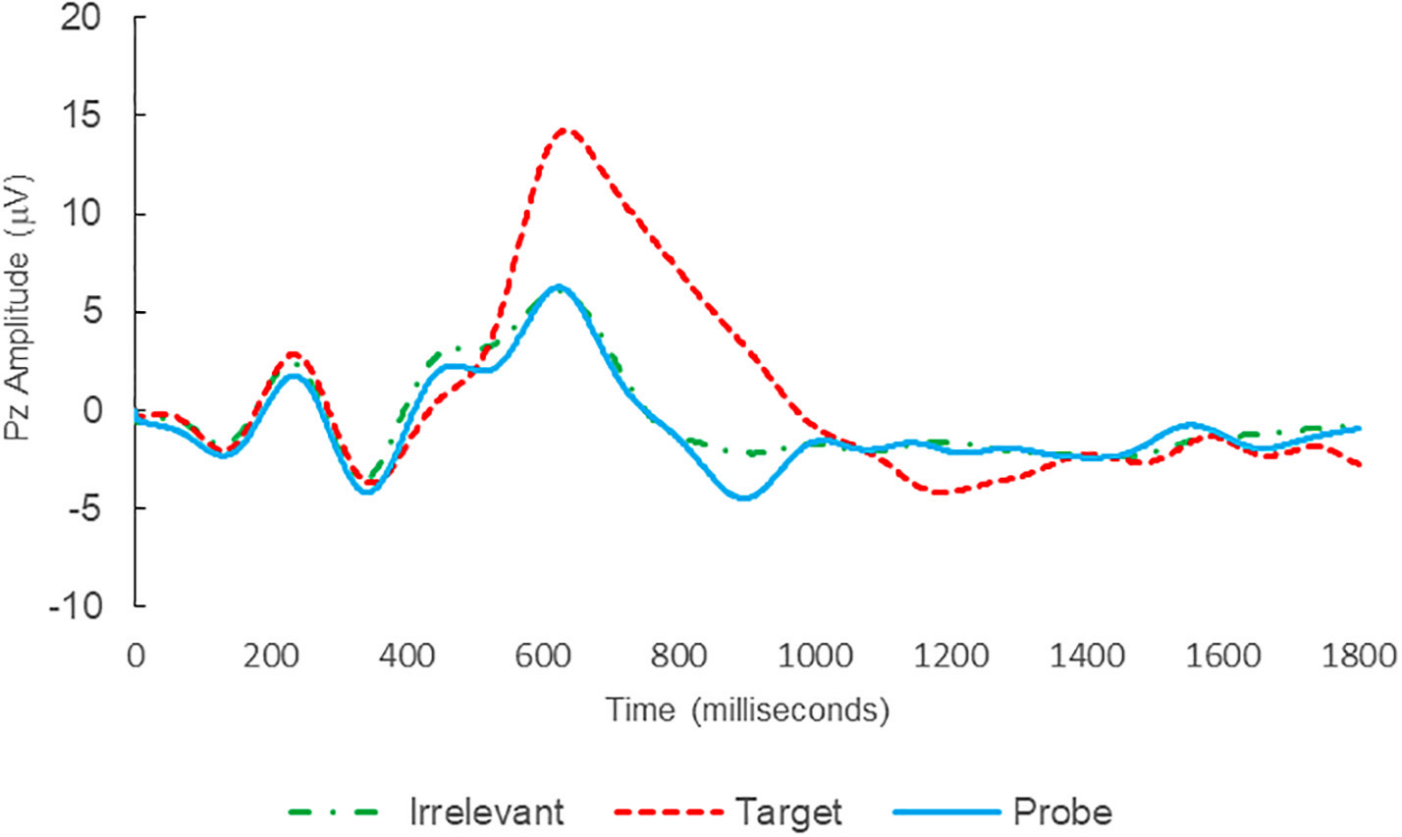
BFP response waveforms of C03 in “Flatmate Assault” (IA → IA_C_).

**FIGURE 7:**
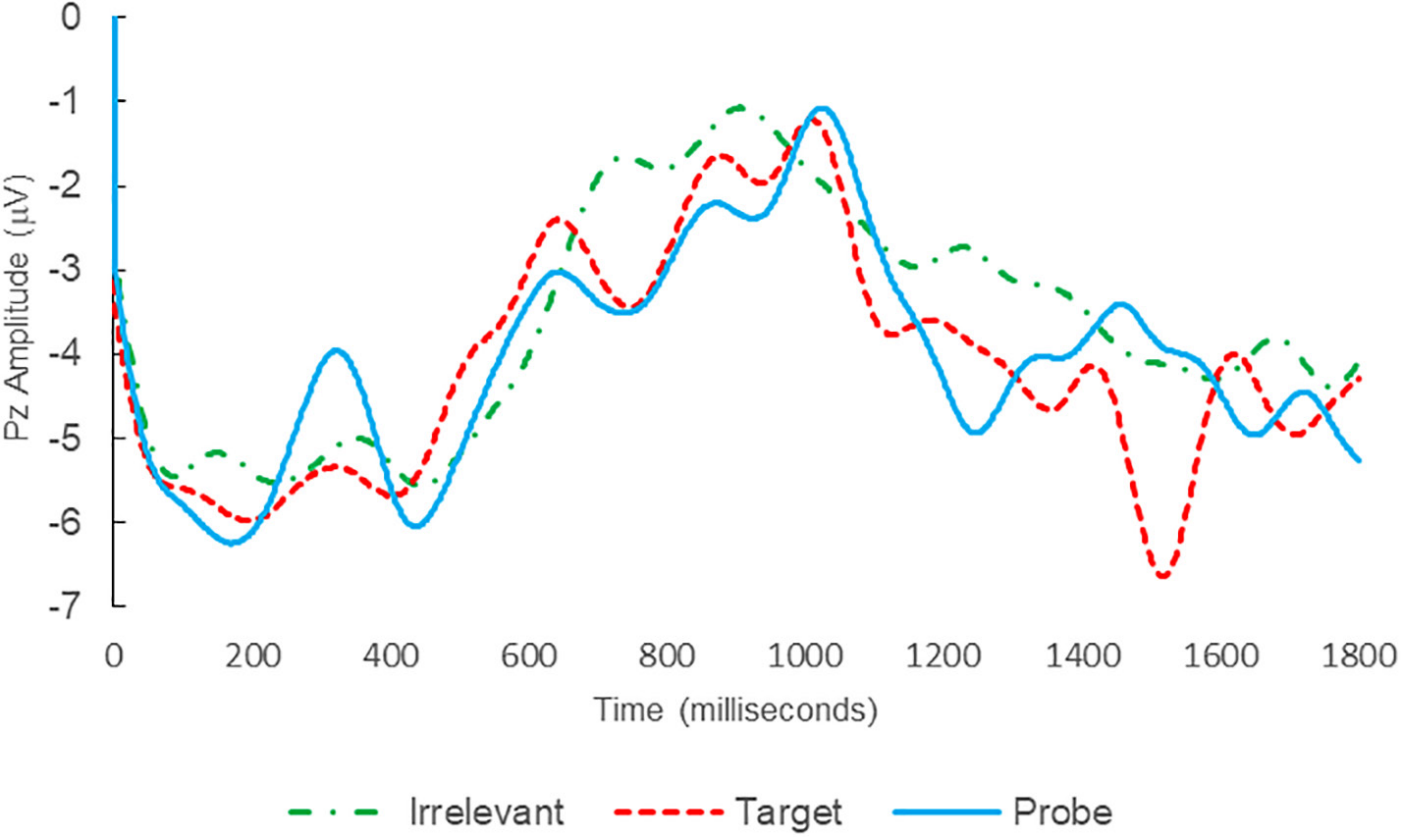
BFP response waveforms of C07 in “Armour Guard Heist” (IP → IP_C_).

**FIGURE 8:**
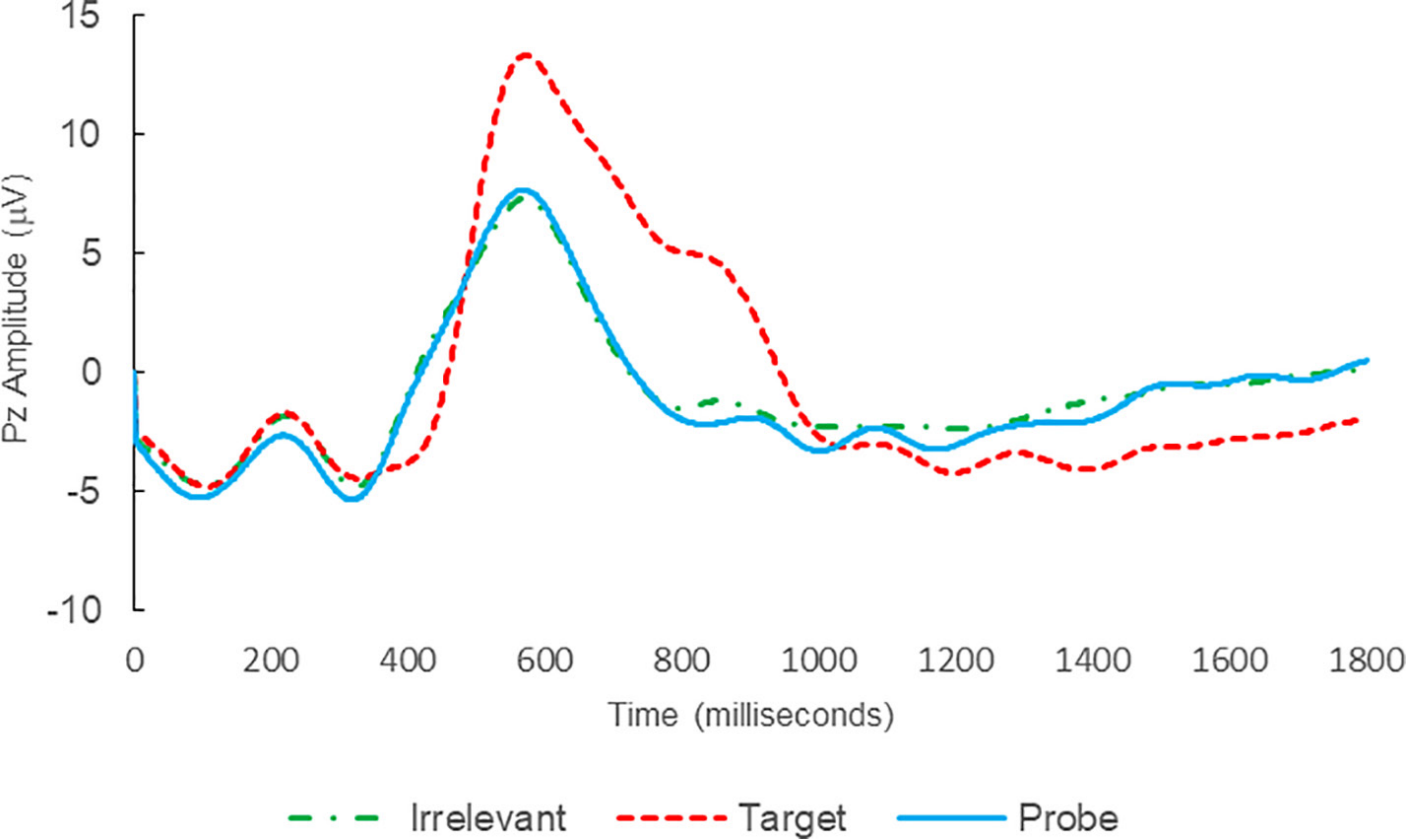
BFP response waveforms of C08 in “Armour Guard Heist” (IA → IA_C_).

**FIGURE 9:**
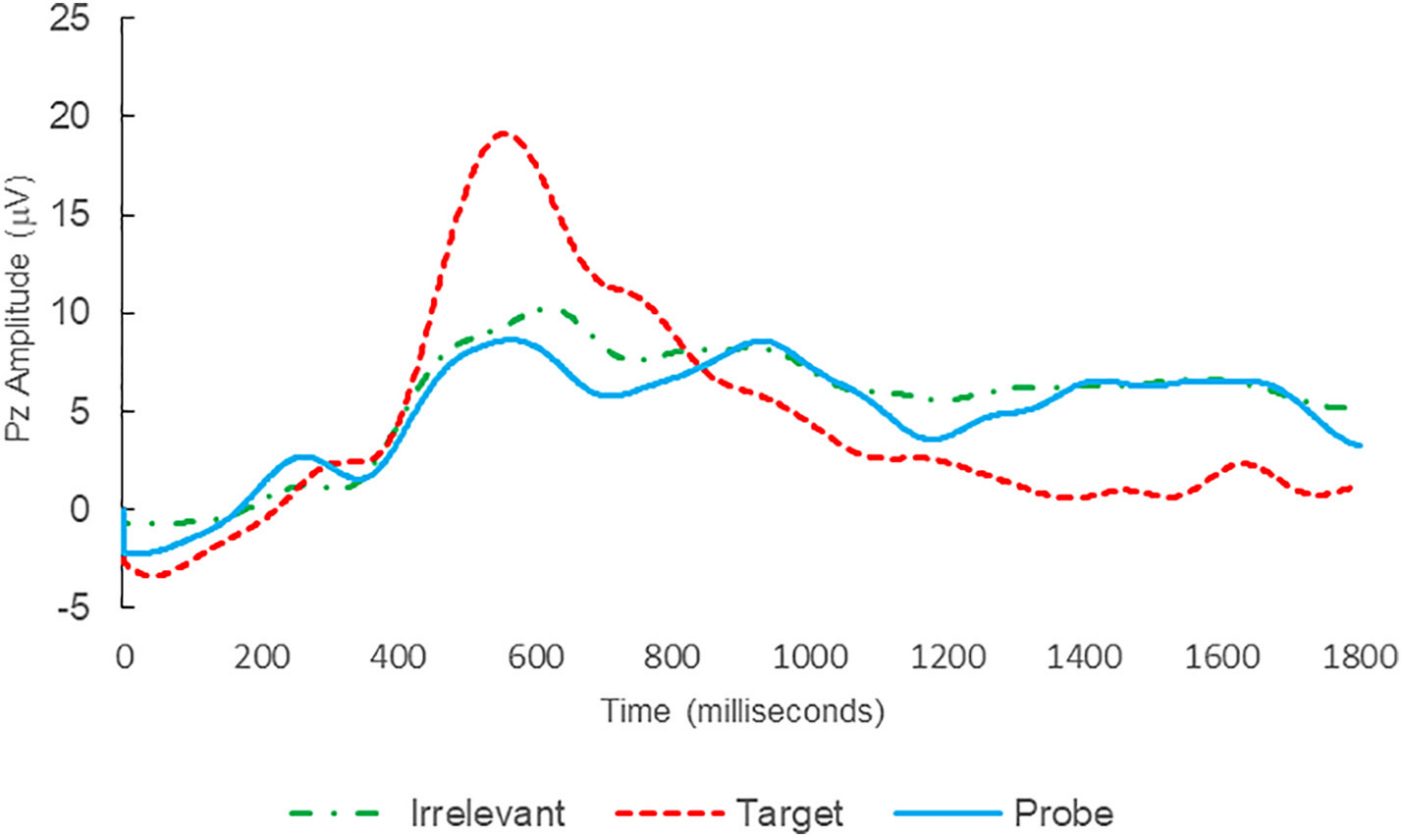
BFP response waveforms of C09 in “Armour Guard Heist” (IA → IA_C_).

**FIGURE 10:**
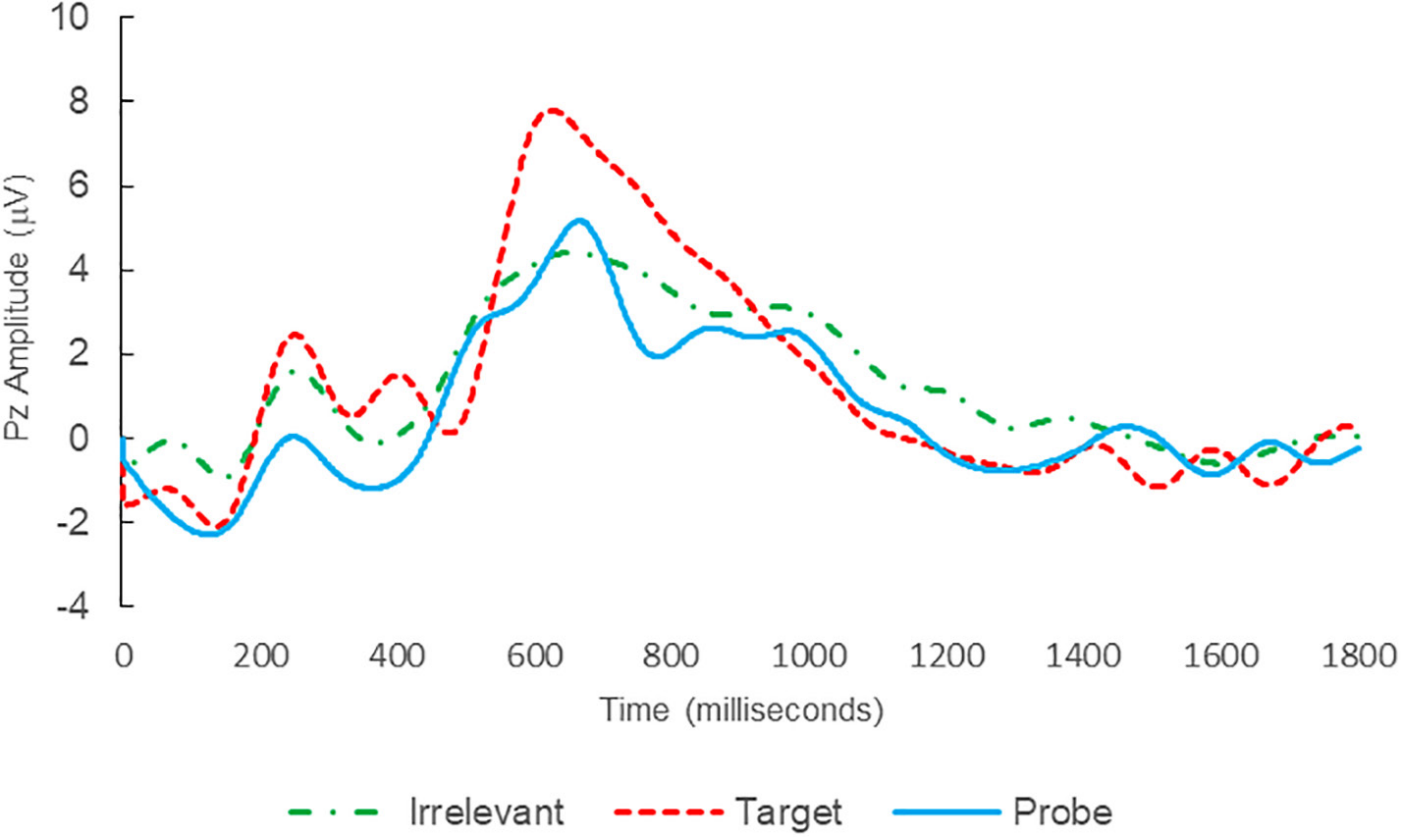
BFP response waveforms of C10 in “Armour Guard Heist” (IA → Indeterminate).

**FIGURE 11:**
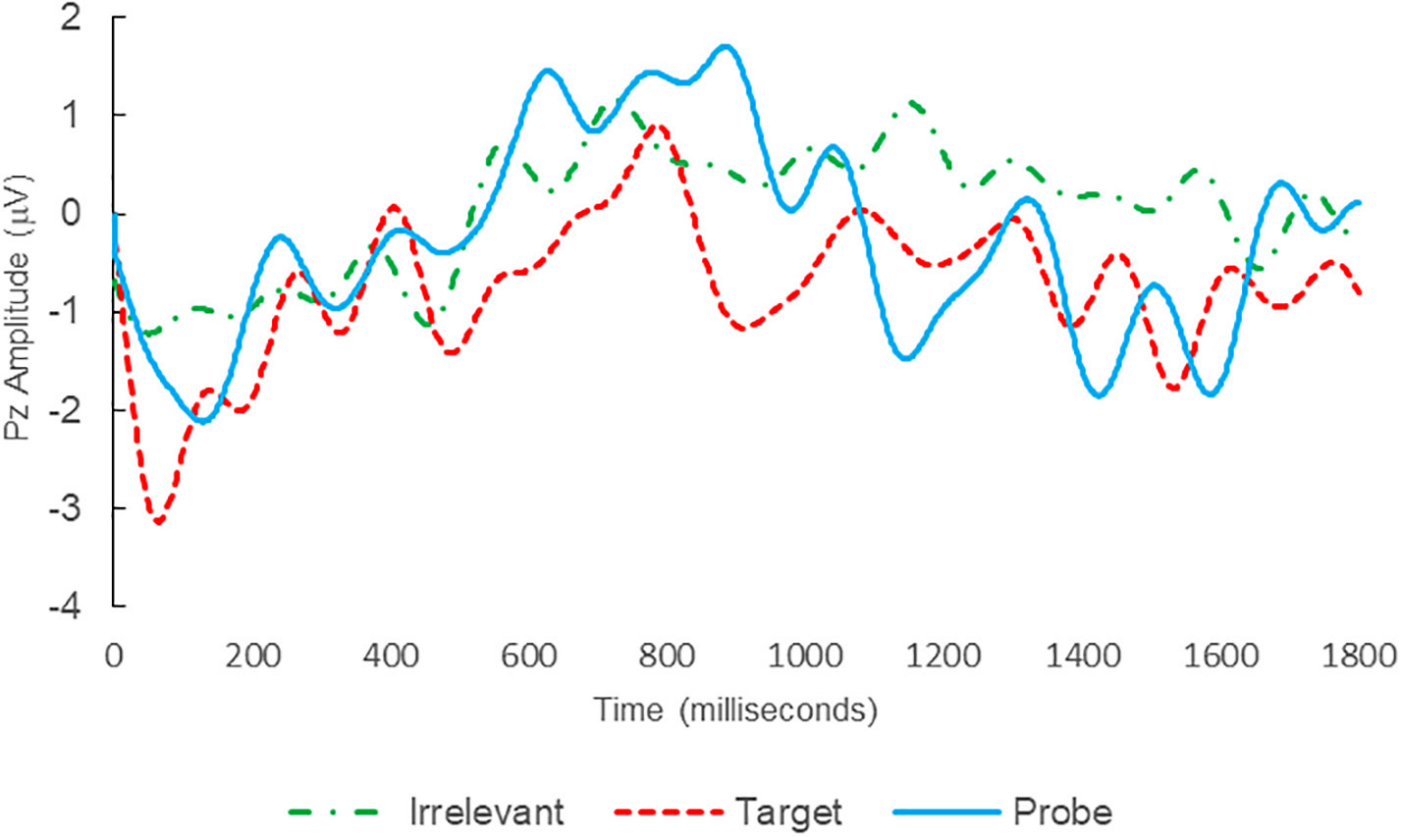
BFP response waveforms of C11 in “Armour Guard Heist” (IA → IP_C_).

**FIGURE 12:**
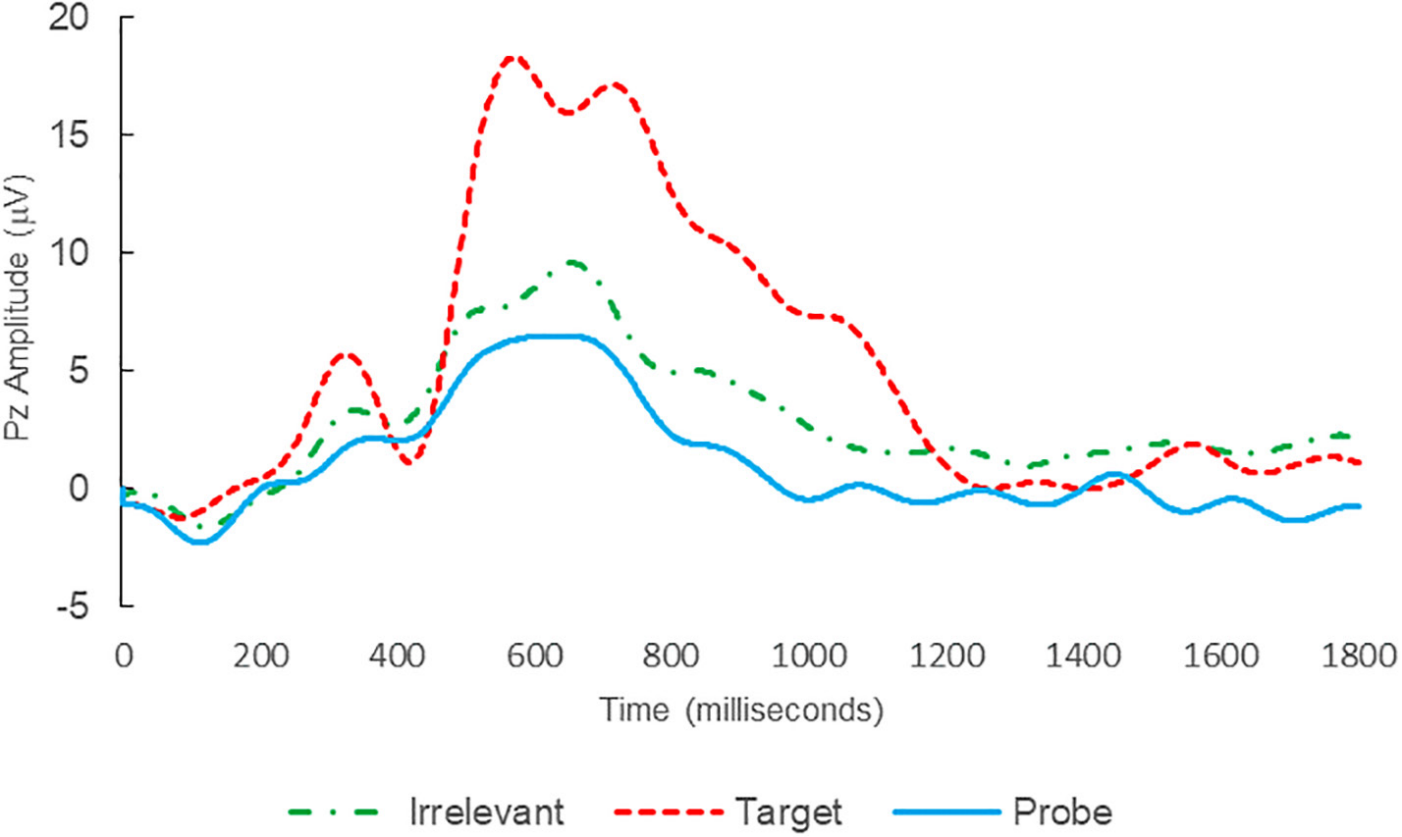
BFP response waveforms of C13 in “Robbery” (IA → IA_C_).

**FIGURE 13:**
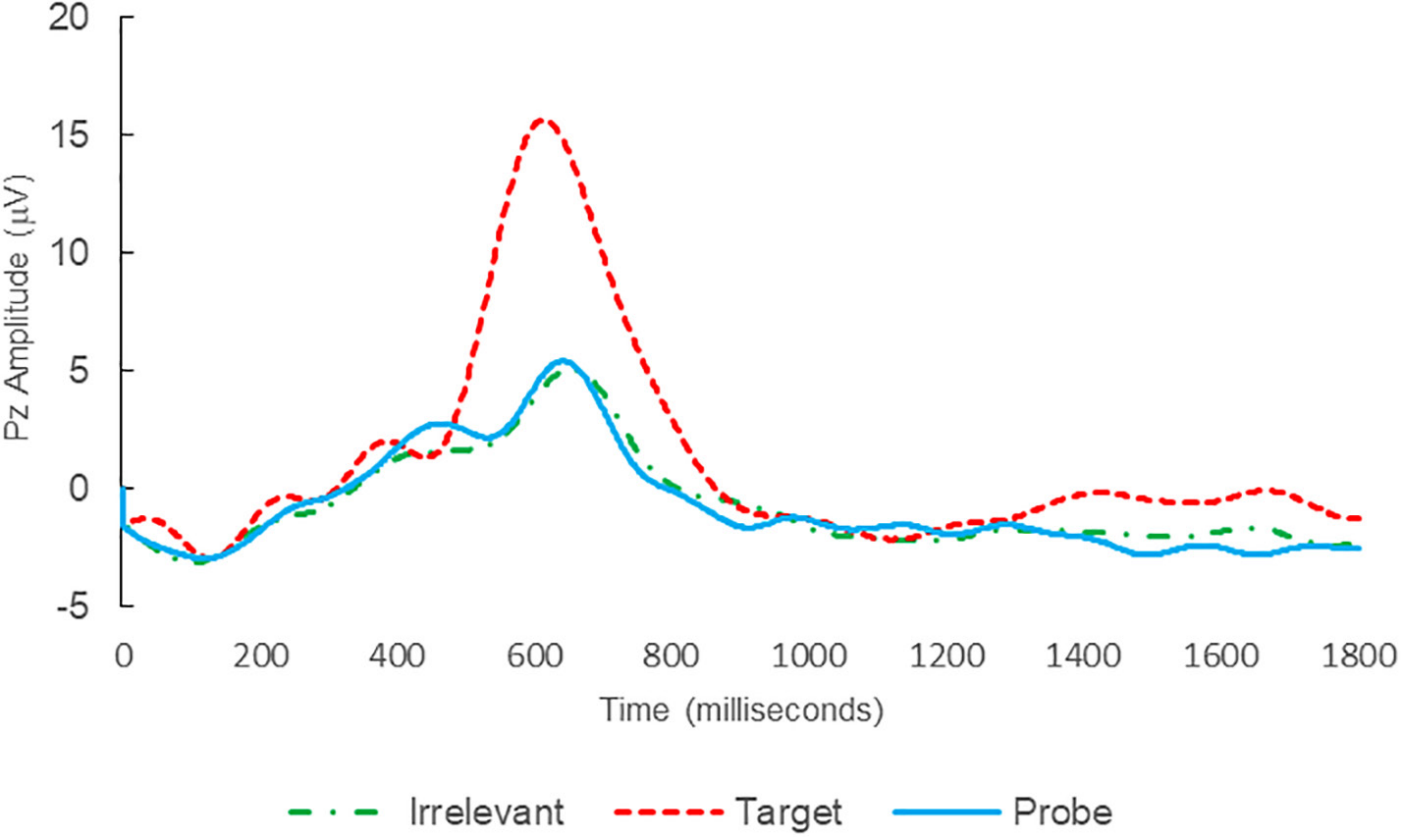
BFP response waveforms of C14 in “Robbery” (IA → IA_C_).

**FIGURE 14:**
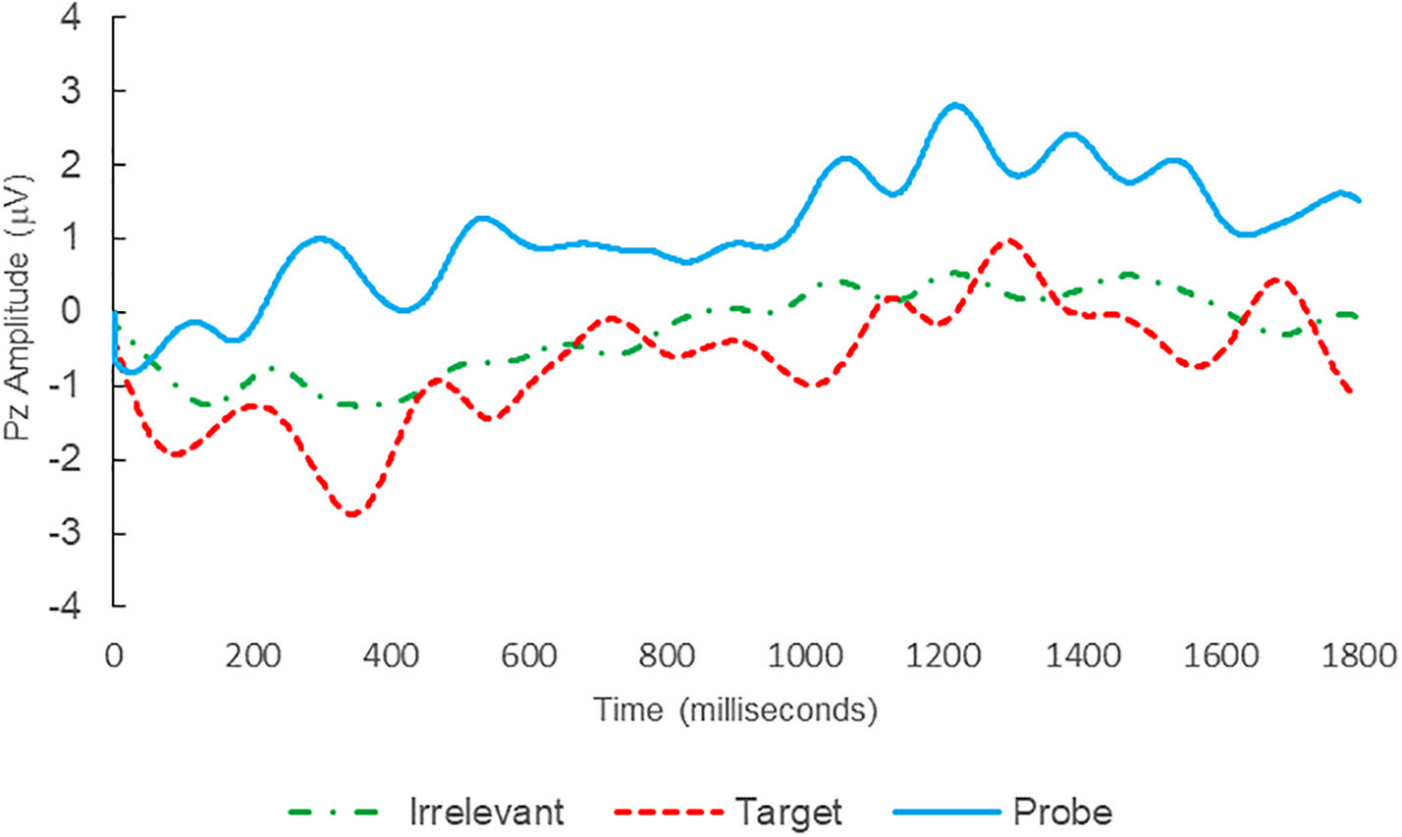
BFP response waveforms of C15 in “Robbery” (IA → Indeterminate).

**FIGURE 15:**
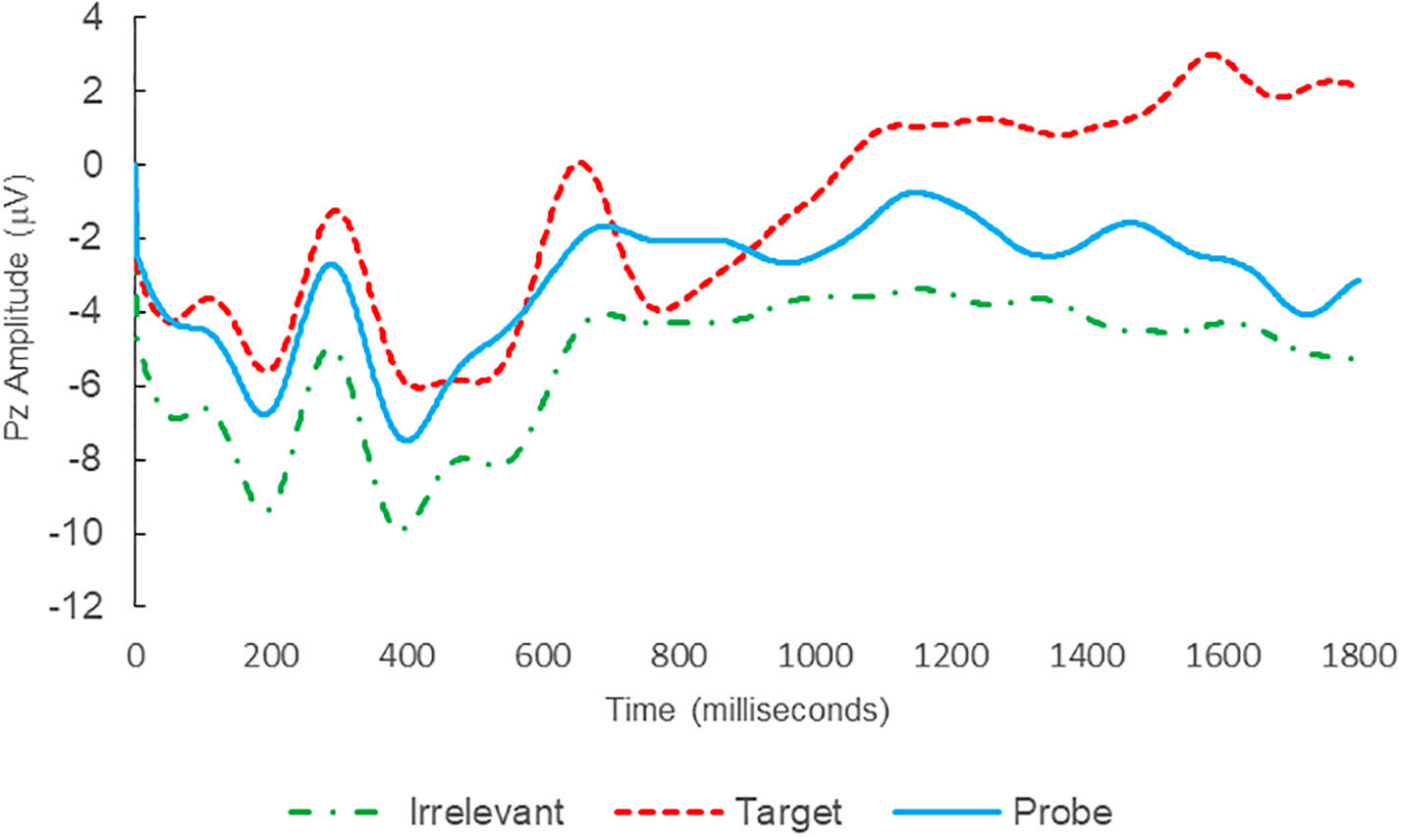
BFP response waveforms of C16 in “Robbery” (IA → Indeterminate).

Of the 12 remaining subjects in Study 2, both IP subjects were correctly classified as IP_C_, with a mean bootstrapping probability of 98.8%, and six of 10 IA subjects were correctly classified as IA_C_, with a mean bootstrapping probability of 99.4%. One IA subject (C11) was misclassified as IP_C_, with a bootstrapping probability of 93.5%, and three IA subjects (C10, C15, and C16) were Indeterminates (see Table 4 and Figures 4, 5, 6, 7, 8, 9, 10, 11, 12, 13, 14, 15 for ERPs).

**TABLE 4:** Summary of Brain Fingerprinting results for study 2

Incident	Subject ID	Ground‐ truth	BFP determination	Bootstrapping probability (%)
Flatmate Assault	C01	IP	IP_C_	99.9
	C02	IA	IA_C_	92.6
	C03	IA	IA_C_	99.9
	C04	IA	Excluded (<100 trials)	
	C05	IA	Withdrew due to eye‐fatigue	
	C06	IA	Withdrew due to eye‐fatigue	
Armour Guard Heist	C07	IP	IP_C_	98.5
	C08	IA	IA_C_	98.4
	C09	IA	IA_C_	98.7
	C10	IA	IND ^a^	53.7
	C11	IA	IP_C_ ^b^	93.5
Robbery	C12	IP	Withdrew due to eye‐fatigue	
	C13	IA	IA_C_	99.9
	C14	IA	IA_C_	99.7
	C15	IA	IND ^a^	67.5
	C16	IA	IND ^a^	56.2
Revenge	C17	IP	Never participated	

*Note*: The letter “C” preceding each subject number refers to “Real‐Crime” incidents.

Abbreviations: BFP, Brain Fingerprinting; IA, ground‐truth Information‐Absent; IA_C_, classified as Information‐Absent by Brain Fingerprinting; IND, classified as Indeterminate by Brain Fingerprinting; IP, ground‐truth Information‐Present; IP_C_, classified as Information‐Present by Brain Fingerprinting.

^a^ Blue colored font shows an Indeterminate classification.

^b^ Red colored font shows a false positive classification.

Thus, eight out of 12 classifications were correct (accuracy of 91.7% discounting Indeterminates), and consistent with ground‐truth, with one false positive, three Indeterminates and no false negatives, with a mean bootstrap probability for correct determinations of 99.3%.

#### 3.2.3 Further analysis of C11

**FIGURE 16:**
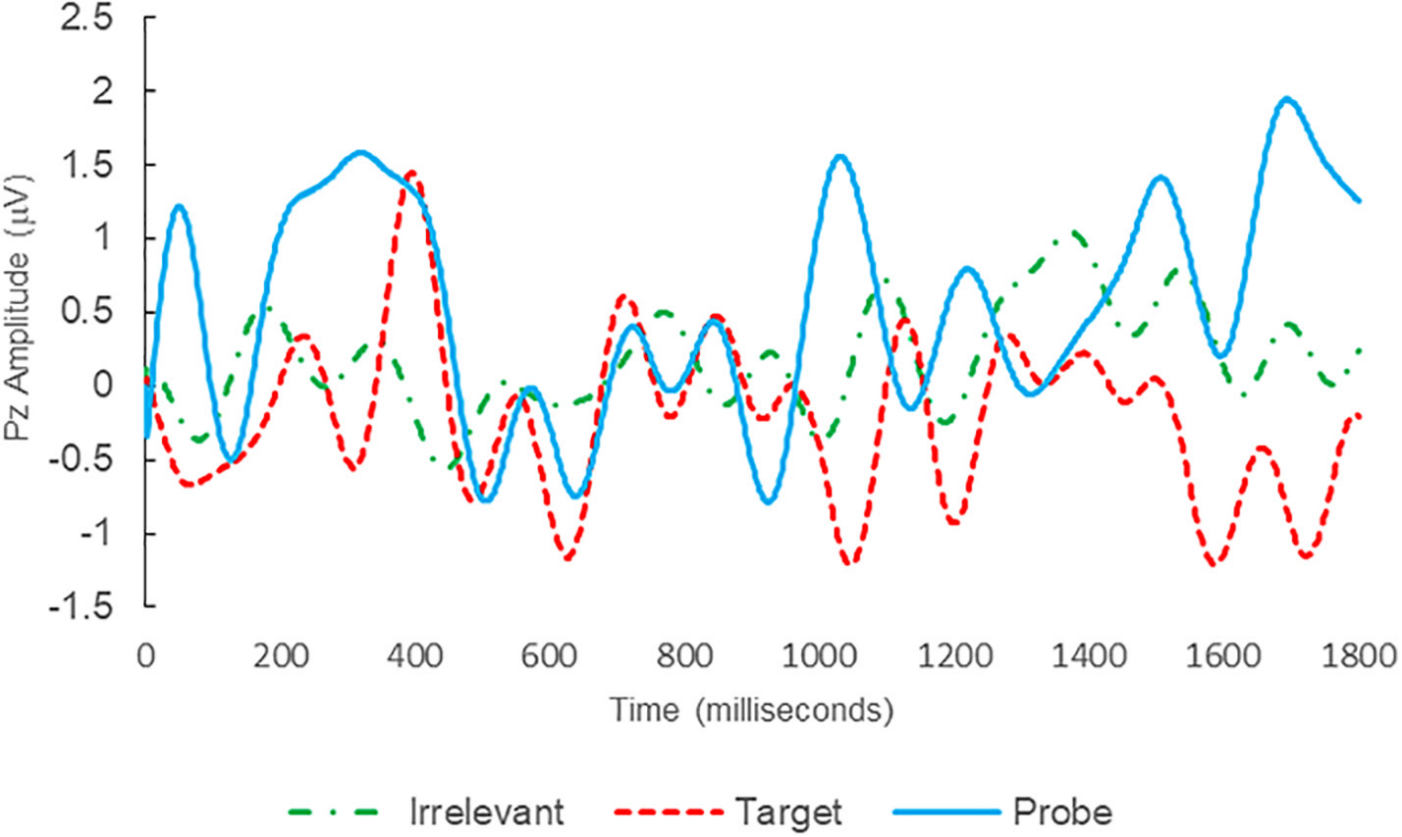
BFP response waveforms of C11 in “Robbery” (IA → IP_C_).

**FIGURE 17:**
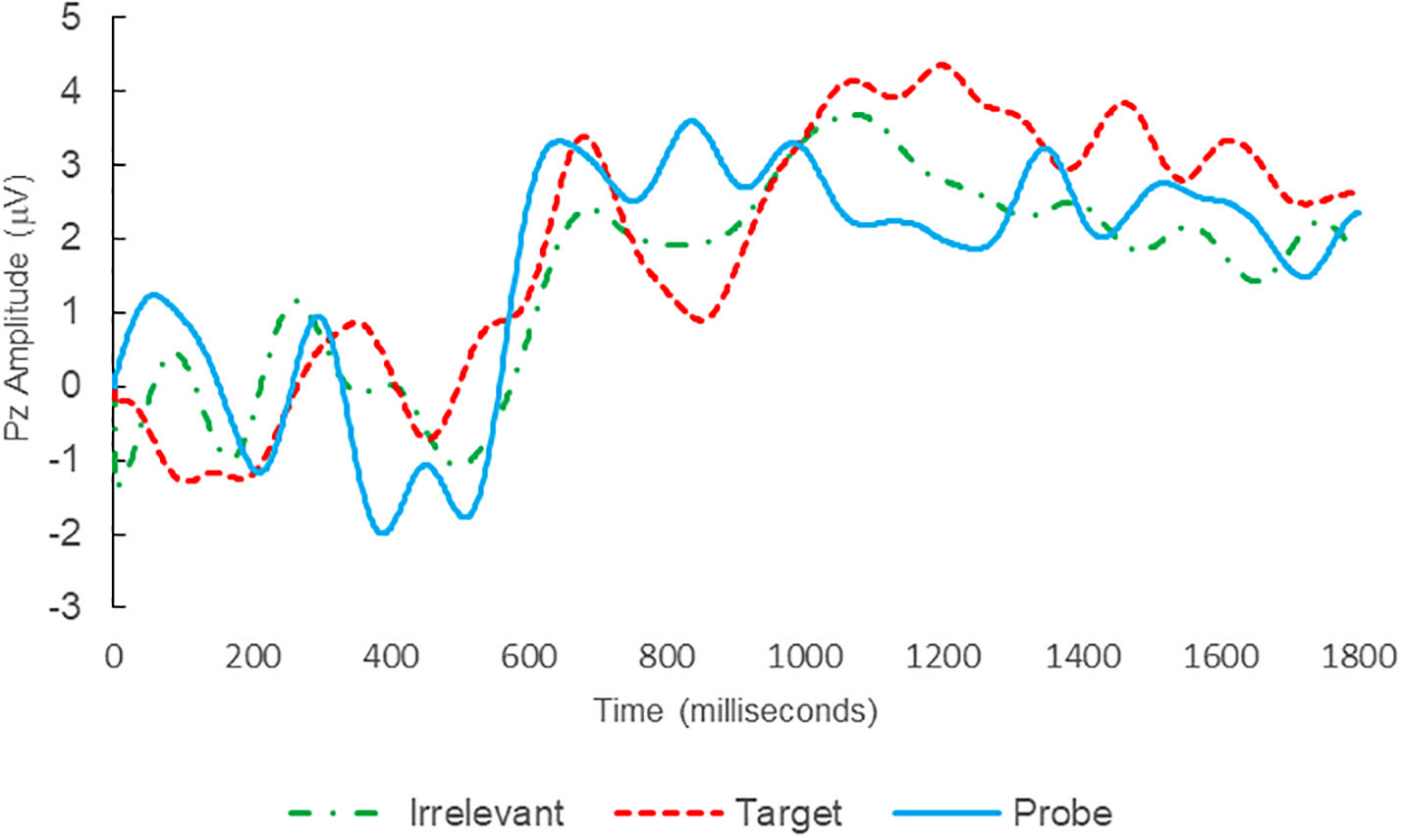
BFP response waveforms of C11 in “Stolen Dog” (IP → Indeterminate).

As IA subject C11 in Study 2 was incorrectly classified as IP_C_ for the *Armor Guard Heist* incident, we decided, with his willing consent, to investigate him further. He was retested on the *Robbery* incident for which he was also IA and for which he was again incorrectly determined as IP_C_. We subsequently tested him on his own crime incident: the *Stolen Dog*. Although now IP, he was classified by BFP as Indeterminate. See Table 5 for further details and Figures 16 and 17 for his ERPs for the IA and IP incidents.

**TABLE 5:** C11's BFP determination for different incidents

Incident	Ground‐truth	BFP determination	Bootstrapping probability (%)
Armour Guard Heist	IA	IP_C_ ^a^	93.5
Robbery	IA	IP_C_ ^a^	90.1
Stolen Dog	IP	IND ^b^	72.6

Abbreviations: BFP, Brain Fingerprinting; IA, ground‐truth Information‐Absent; IND, classified as Indeterminate by Brain Fingerprinting; IP, ground‐truth Information‐Present; IP_C_, classified as Information‐Present by Brain Fingerprinting.

^a^ Red colored font shows a false positive classification.

^b^ Blue colored font shows an Indeterminate classification.

#### 3.2.4 Behavioral accuracy

All subjects in both studies met the behavioral accuracy criteria: each individual block met the accuracy criterion such that accuracies of target and irrelevant stimuli were above 80%. We compared the behavioral accuracy of eight correctly classified subjects of Study 2 with the four classified otherwise (three Indeterminates and one false positive). The median behavioral accuracy of the correctly classified group (*Med* = 94.6%) was seemingly higher than those classified otherwise (*Med* = 80.3%). However, the Mann–Whitney *U* test showed that these differences were not significant, *U* = 8.00, *p* = .214. We also obtained the ratio of discarded trials due to artifacts and compared these two groups. The “otherwise classified” group had a significantly smaller median for analyzed trials (*Med* = 67.5%) than the correctly classified group (*Med* = 90.8%). These differences were significant, *U* = 2.00, *p* = .016, showing that the otherwise classified subjects had a larger ratio of discarded trials than the correctly classified group.

## 4 DISCUSSION

These two studies are the first independent replication evaluations of Brain Fingerprinting. Brain Fingerprinting was used to determine the presence or absence of concealed knowledge in 28 student subjects in Study 1 and in 12 parolees in Study 2. This is also the first project to have tested convicted criminals on their own crime incidents, rather than suspects or convicted criminals claiming innocence (e.g., Farwell et al., 2013), using a forensic brainwave analysis tool.

We have demonstrated that Brain Fingerprinting testing is independently reproducible with high accuracy in a non‐field context. However, our findings do not corroborate the claims of 100% accuracy made by BFP proponents. We found that two IA subjects (possessing no knowledge of the incident on which they were tested) were erroneously determined IP_C_ (classified as possessing knowledge of the incident), one in Study 1 and one in Study 2. Both subjects were cooperative, had high behavioral accuracies (i.e., were appropriately concentrating and correctly performing behavioral recognition of the probe, target, and irrelevant stimuli), and met all 20SS. Furthermore, the same misclassification was repeated when one of these subjects (C11), still as an IA, was tested on a second crime incident. Further enquiries confirmed that neither subject had been aware of these incidents. These findings are inconsistent with previous BFP research (Farwell et al., 2013, 2014; Farwell & Donchin, 1991; Farwell & Smith, 2001) in which BFP testing has reportedly produced zero classification errors, and our findings constitute the first documented instance of BFP misclassifications.

The present results support the notion that ERP‐based measures can achieve high levels of accuracy when both the P300 and LNP are analyzed. BFP analysis utilizing the P300 and late negative potential (“P300‐MERMER”) has produced 0% errors and 0% Indeterminates in previous studies (Farwell et al., 2013, 2014). In contrast, previous BFP research utilizing the P300 only has demonstrated similar accuracy but resulted in some Indeterminates (Farwell & Donchin, 1991). The present studies, analyzing the entire P300‐MERMER epoch, resulted in one false positive in each of two studies, as well as three Indeterminates in Study 2. Our findings demonstrate that, subject to close adherence to 20SS, accurate detection of concealed information by Brain Fingerprinting can be achieved. However, our findings do not support claims from previous studies that BFP has no false positives and no Indeterminates (Farwell, 2009, 2012; Farwell et al., 2013; Farwell & Makeig, 2019). Therefore, our research hypothesis has not been supported. Notwithstanding, both of our studies support claims that BFP has no false negatives.

Iacono (2008) stated that Guilty Knowledge Tests have false positive rates of 2%–5% but that this is common for other types of incriminating tests too. It has also been established that, even when administered properly, psychophysiological memory detection techniques can result in false positives and that the scientific community still values P300‐based tests over autonomic measures of guilt detection (Iacono, 2008).

It is notable that none of the former BFP studies (Farwell et al., 2013, 2014; Farwell & Donchin, 1991; Farwell & Smith, 2001) reported subjects unable to satisfactorily complete the BFP test. However, we had three such subjects in Study 2, all of whom could not complete BFP due to inability to control excessive blinking and/or physical jitteriness. This is important as it reveals that some people are simply unable to satisfactorily meet the requirements of the BFP test.

Optimal signal conditioning is very important in ERP studies. Thus, poor/noisy EEG from some of our subjects is likely to be the cause of at least some of the unexpected classifications. A particularly striking example of this is Subject C11 in Study 2. Notwithstanding, it is difficult to explain why he was not classified as Indeterminate, nor how such aberrant‐looking ERPs could be gained in three separate BFP tests from a subject who was so compliant and had no problem with excessive blinking or keeping still.

There is a notable difference between the Studies 1 and 2 in terms of Indeterminate classifications (0 and 3, respectively). It would appear that this reflects differences between the two study cohorts of university students versus parolees, although whether this, in turn, is a reflection of time in prison, personality, life‐style, genetic, or other differences is unknown. It is also worth noting that the correctly classified subjects in Study 2 had fewer discarded trials than other subjects. However, the behavioral accuracies were not significantly different across Study 2 subjects.

Further independent research designed to improve upon the relatively low ecological validity of the present study by utilizing a subject pool more representative of BFP's primary application target in the forensic field—that is, crime suspects and persons convicted but maintaining their claim of innocence—would be beneficial. The small number of parolees—especially only two IPs—was a limitation of Study 2. However, we wish to emphasize that, unlike previous BFP studies, the subjects in this study were real‐life convicted criminals. We found it challenging to find and recruit parolees with dangerous crime histories who were willing to participate in tests on their own crime incidents and be compliant with experimental protocols. Despite these difficulties, we consider Study 2 was an important step toward determining BFP's efficacy, limitations, and potential in the forensic field.

There is another dimension in the testing of parolees. It might be considered that there is little value in determining the presence of information in criminals who have already confessed, completed their time in prison, and for which there are no further consequences. We recognize this but, as BFP has not been established as a robust forensic tool, this population was chosen due to their possessing many of the sociopathological, psychopathological, life‐style, and other characteristic traits of the suspects and criminals for whom forensic brainwave analysis will be a primary target.

### 4.1 Limitations

We previously mentioned the small sample size in Study 2 as a limitation. It should also be acknowledged that the BFP software does not provide data on baseline correction, eye‐movement correction, correction for amplifier drift, flatlining, pre‐stimulus activity, etc. We suggest that the algorithm should be improved so these data can also be analyzed and reported in future studies.

It must be emphasized that the current studies were conducted with a test protocol which is critically different to that needed in real‐world application of BFP/FBA. In the latter crime‐detection setting, information would be gathered from a crime scene and the critical stimuli formulated based on that (i.e., not narrated to the testers by a subject). This also means that the *information confirmation* procedure would not be needed or valid in a real‐life setting. Once the BFP test stimuli and questions are developed, all suspects are examined and BFP determinations of their IP or IA statuses are made. Notwithstanding, the stories told to the testers by Study 2 subjects were corroborated with the police records to ensure the accuracy of incidents. These were real‐crime incidents but the procedure was kept similar with Study 1. Real‐life crime‐detection testing using information directly from the crime scene or the criminal crime records could be conducted in the future once the current BFP protocol is well established and the previously mentioned limitations are addressed.

Former BFP studies, including Farwell et al. (2013), promised incentive to their subjects in case they could render the BFP ineffective, or they were motivated because of facing legal consequences due to criminal offenses. It could be considered a limitation that our subjects did not have such high motivations. However, the fact that these subjects were happy to volunteer to participate shows that they were motivated to participate and follow the instructions. Provision of further motivation to subjects will be considered in future studies. Notwithstanding, in real‐world forensic applications, BFP's classification needs to be independent of a subject's level of motivation; achievement of an acceptable level of behavioral accuracy should be all that is required for BFP accuracy to be valid.

Lastly, another limitation in Study 2 was that, following subject withdrawals and exclusions, there was a substantial imbalance between the two IP subjects and the 10 IA subjects.

## 5 CONCLUSION

The “Brain Fingerprinting” technique is built on a solid theoretical framework of phenomena that have been widely supported in the scientific literature. However, the efficacy of Brain Fingerprinting has been cast into doubt, in part due to a lack of independent validation of the technique. The present two independent studies demonstrate high accuracy in detection of concealed knowledge with BFP in a non‐field context. However, our findings do not corroborate the 100% accuracy achieved by prior (non‐independent) research. The present findings also include the first documented false positives and Indeterminates in the literature of Brain Fingerprinting with the full 1800‐ms ERP (“P300‐MERMER”), despite rigorous adherence to the 20 Brain Fingerprinting Scientific Standards. In addition, we also identified that Brain Fingerprinting is not a viable test for everyone, especially persons unable to suppress excessive eye‐blinking. Notwithstanding, it is important to note that there were no false negatives.

Further investigation using forensic contexts would lend more clarity regarding the accuracy and applicability of Brain Fingerprinting testing. Overall, we conclude that BFP is not yet at a stage at which it can be used as a robust and completely accurate crime‐detection tool. However, we suggest that forensic brainwave research should continue in order to address the problems we have identified, as this technology has the potential to be developed into a powerful new tool in forensic investigations and related applications.

## AUTHOR CONTRIBUTIONS

**Usman Afzali:** Conceptualization; data curation; formal analysis; investigation; methodology; visualization; writing – original draft; writing – review and editing. **Alex Seren‐Grace:** Data curation, formal analysis, investigation, methodology, writing – original draft, writing – review & editing. **Robin Palmer:** Conceptualization; data curation; formal analysis; funding acquisition; investigation; methodology; project administration; supervision; writing – review and editing. **Ewald Neumann:** Conceptualization; investigation; methodology; supervision; writing – review and editing. **Sarah Makarious:** Data curation; formal analysis; writing – review and editing. **Debra Wilson:** Conceptualization; methodology; writing – review and editing. **Richard Jones:** Conceptualization; investigation; methodology; supervision; writing – review and editing.

## CONFLICT OF INTEREST

We have no conflict of interest to disclose.

## Supporting information


**Appendix** The Brain Fingerprinting Scientific Standards

## References

[psyp14110-bib-0001] Allen, J. J. , Iacono, W. G. , & Danielson, K. D. (1992). The identification of concealed memories using the event‐related potential and implicit behavioral measures: A methodology for prediction in the face of individual differences. Psychophysiology, 29(5), 504–522. 10.1111/j.1469-8986.1992.tb02024.x 1410180

[psyp14110-bib-0002] Allen, J. J. B. , & Iacono, W. G. (1997). A comparison of methods for the analysis of event‐related potentials in deception detection. Psychophysiology, 34(2), 234–240. 10.1111/j.1469-8986.1997.tb02137.x 9090275

[psyp14110-bib-0003] Ben‐Shakhar, G. (2012). Current research and potential applications of the concealed information test: An overview. Frontiers in Psychology, 3, 1–11. 10.3389/fpsyg.2012.00342 23060826 PMC3462434

[psyp14110-bib-0004] Ben‐Shakhar, G. , & Elaad, E. (2003). The validity of psychophysiological detection of information with the guilty knowledge test: A meta‐analytic review. Journal of Applied Psychology, 88(1), 131–151. 10.1037/0021-9010.88.1.131 12675401

[psyp14110-bib-0005] Bergström, Z. M. , Anderson, M. C. , Buda, M. , Simons, J. S. , & Richardson‐Klavehn, A. (2013). Intentional retrieval suppression can conceal guilty knowledge in ERP memory detection tests. Biological Psychology, 94(1), 1–11. 10.1016/j.biopsycho.2013.04.012 23664804 PMC3749379

[psyp14110-bib-0006] Berlad, I. , & Pratt, H. (1995). P300 in response to the subject's own name. Electroencephalography and Clinical Neurophysiology/Evoked Potentials Section, 96(5), 472–474. 10.1016/0168-5597(95)00116-A 7555920

[psyp14110-bib-0007] Donchin, B. , Karis, D. , Bashore, T. R. , Coles, M. G. H. , & Gratton, G. (1986). Cognitive psychophysiology and human information processing. In M. G. H. Coles , E. Donchin , & S. Porges (Eds.), Psychophysiology: Systems, processes, and applications (pp. 244–267). Guilford Press.

[psyp14110-bib-0008] Farwell, L. A. (2009). Brain fingerprinting: Detection of concealed information. Wiley Encyclopedia of Forensic Science, 1–12. 10.1002/9780470061589.fsa1013

[psyp14110-bib-0009] Farwell, L. A. (2011). Brain fingerprinting: Corrections to Rosenfeld. Scientific Review of Mental Health Practice, 8(2), 46–68.

[psyp14110-bib-0010] Farwell, L. A. (2012). Brain fingerprinting: A comprehensive tutorial review of detection of concealed information with event‐related brain potentials. Cognitive Neurodynamics, 6(2), 115–154. 10.1007/s11571-012-9192-2 23542949 PMC3311838

[psyp14110-bib-0011] Farwell, L. A. , & Donchin, E. (1991). The truth will out: Interrogative polygraphy (“Lie Detection”) with Event‐Related Brain Potentials. Psychophysiology, 28(5), 531–547. 10.1111/j.1469-8986.1991.tb01990.x 1758929

[psyp14110-bib-0012] Farwell, L. A. , & Makeig, T. H. (2019). Farwell brain fingerprinting in the case of Harrington v. State. Farwell Brain Fingerprinting ‐ Dr. Larry Farwell. http://www.larryfarwell.com/pdf/OpenCourtFarwellMakeig‐dr‐larry‐farwell‐brain‐fingerprinting‐dr‐lawrence‐farwell.pdf

[psyp14110-bib-0013] Farwell, L. A. , & Richardson, D. C. (2013). Brain fingerprinting: Let's focus on the science ‐ A reply to Meijer, Ben‐Shakhar, Verschuere, and Donchin. Cognitive Neurodynamics, 7(2), 159–166. 10.1007/s11571-012-9238-5 23494087 PMC3595431

[psyp14110-bib-0014] Farwell, L. A. , Richardson, D. C. , & Richardson, G. M. (2013). Brain fingerprinting field studies comparing P300‐MERMER and P300 brainwave responses in the detection of concealed information. Cognitive Neurodynamics, 7(4), 263–299. 10.1007/s11571-012-9230-0 23869200 PMC3713201

[psyp14110-bib-0015] Farwell, L. A. , Richardson, D. C. , Richardson, G. M. , & Furedy, J. J. (2014). Brain fingerprinting classification concealed information test detects US Navy military medical information with P300. Frontiers in Neuroscience, 8, 1–21. 10.3389/fnins.2014.00410 25565941 PMC4274905

[psyp14110-bib-0016] Farwell, L. A. , & Smith, S. S. (2001). Using brain MERMER testing to detect knowledge despite efforts to conceal. Journal of Forensic Science, 46(1), 135–143. 10.1520/JFS14925J 11210899

[psyp14110-bib-0017] Funicelli, M. , White, L. , Ungureanu, S. , & Laurence, J.‐R. (2021). An independent validation of the EEG‐based complex trial protocol with autobiographical data and corroboration of its resistance to a cognitively charged countermeasure. Applied Psychophysiology and Biofeedback, 46, 287–299. 10.1007/s10484-021-09506-2 33655464

[psyp14110-bib-0018] Gamer, M. (2011). Detecting concealed information using autonomic measures. In G. B.‐S. B. Verschuere & E. Meijer (Eds.), Memory detection: Theory and application of the concealed information test (pp. 27–45). Cambridge University Press. 10.1017/CBO9780511975196.003

[psyp14110-bib-0019] Gray, H. M. , Ambady, N. , Lowenthal, W. T. , & Deldin, P. (2004). P300 as an index of attention to self‐relevant stimuli. Journal of Experimental Social Psychology, 40(2), 216–224. 10.1016/S0022-1031(03)00092-1

[psyp14110-bib-0020] Iacono, W. G. (2008). The forensic application of “brain fingerprinting:” Why scientists should encourage the use of P300 memory detection methods. The American Journal of Bioethics, 8(1), 30–32. 10.1080/15265160701828550 18236333

[psyp14110-bib-0021] Johnson, D. T. (Ed.). (2020). Wrongful convictions and the culture of denial. In The culture of capital punishment in Japan (pp. 61–80). Springer.

[psyp14110-bib-0022] Johnson, R. (1986). A triarchic model of P300 amplitude. Psychophysiology, 23(4), 367–384. 10.1111/j.1469-8986.1986.tb00649.x 3774922

[psyp14110-bib-0023] klein Selle, N. , Waxman, D. , Volz, K. , Ambach, W. , & Ben‐Shakhar, G. (2021). Is the CIT susceptible to misleading information? A constructive replication. Journal of Forensic Sciences, 66(2), 646–655. 10.1111/1556-4029.14630 33227162

[psyp14110-bib-0024] Lukács, G. , Weiss, B. , Dalos, V. D. , Kilencz, T. , Tudja, S. , & Csifcsák, G. (2016). The first independent study on the complex trial protocol version of the P300‐based concealed information test: Corroboration of previous findings and highlights on vulnerabilities. International Journal of Psychophysiology, 110, 56–65. 10.1016/j.ijpsycho.2016.10.010 27751782

[psyp14110-bib-0025] Lykken, D. T. (1959). The GSR in the detection of guilt. Journal of Applied Psychology, 43(6), 385–388. 10.1037/h0046060

[psyp14110-bib-0026] Lykken, D. T. (1960). The validity of the guilty knowledge technique: The effects of faking. Journal of Applied Psychology, 44(4), 258–262. 10.1037/h0044413

[psyp14110-bib-0027] MacLaren, V. V. (2001). A quantitative review of the guilty knowledge test. Journal of Applied Psychology, 86(4), 674–683. 10.1037/0021-9010.86.4.674 11519651

[psyp14110-bib-0028] Meijer, E. H. , Ben‐Shakhar, G. , Verschuere, B. , & Donchin, E. (2013). A comment on Farwell (2012): Brain fingerprinting: A comprehensive tutorial review of detection of concealed information with event‐related brain potentials. Cognitive Neurodynamics, 7(2), 155–158. 10.1007/s11571-012-9217-x 23493984 PMC3595430

[psyp14110-bib-0029] Meijer, E. H. , Smulders, F. T. , Merckelbach, H. L. , & Wolf, A. G. (2007). The P300 is sensitive to concealed face recognition. International Journal of Psychophysiology, 66(3), 231–237. 10.1016/j.ijpsycho.2007.08.001 17825933

[psyp14110-bib-0030] Mertens, R. , & Allen, J. J. (2008). The role of psychophysiology in forensic assessments: Deception detection, ERPs, and virtual reality mock crime scenarios. Psychophysiology, 45(2), 286–298. 10.1111/j.1469-8986.2007.00615.x 17995914

[psyp14110-bib-0031] Michigan Law Innocence Clinic . (2021). Causes of wrongful convictions. Michigan Law, University of Michigan. https://www.law.umich.edu/clinical/innocenceclinic/Pages/wrongfulconvictions.aspx

[psyp14110-bib-0032] Moenssens, A. A. (2001). Brain fingerprinting‐can it be used to detect the innocence of persons charged with a crime. UMKC Law Review, 70, 891–920.

[psyp14110-bib-0033] Nose, H. (1981). Gohan no Kenkyu: Nishi Doitsu no Saishin Jirei no Bunseki [translation of Karl Peters,“Fehlerquellen im Strafprozess: Eine Untersuchung der Wiederanfnahmeverfahren in der Bundesrepublik Deutschland,” originally published in 1974]. Hokkaido Daigaku Tosho Kankokai.

[psyp14110-bib-0034] Rosenfeld, J. , Shue, E. , & Singer, E. (2007). Single versus multiple probe blocks of P300‐based concealed information tests for self‐referring versus incidentally obtained information. Biological Psychology, 74(3), 396–404. 10.1016/j.biopsycho.2006.10.002 17126984

[psyp14110-bib-0035] Rosenfeld, J. P. (2005). Brain fingerprinting: A critical analysis. The Scientific Review of Mental Health Practice, 4(1), 20–37.

[psyp14110-bib-0036] Rosenfeld, J. P. (2020). P300 in detecting concealed information and deception: A review. Psychophysiology, 57(7), e13362. 10.1111/psyp.13362 30859600

[psyp14110-bib-0037] Rosenfeld, J. P. , Cantwell, B. , Nasman, V. T. , Wojdac, V. , Ivanov, S. , & Mazzeri, L. (1988). A modified, event‐related potential‐based guilty knowledge test. International Journal of Neuroscience, 42(1–2), 157–161. 10.3109/00207458808985770 3209369

[psyp14110-bib-0038] Rosenfeld, J. P. , & Donchin, E. (2015). Resampling (bootstrapping) the mean: A definite do. Psychophysiology, 52(7), 969–972. 10.1111/psyp.12421 25716059

[psyp14110-bib-0039] Rosenfeld, J. P. , Labkovsky, E. , Winograd, M. , Lui, M. A. , Vandenboom, C. , & Chedid, E. (2008). The Complex Trial Protocol (CTP): A new, countermeasure‐resistant, accurate, P300‐based method for detection of concealed information. Psychophysiology, 45(6), 906–919. 10.1111/j.1469-8986.2008.00708.x 18823418

[psyp14110-bib-0040] Rosenfeld, J. P. , Soskins, M. , Bosh, G. , & Ryan, A. (2004). Simple, effective countermeasures to P300‐based tests of detection of concealed information. Psychophysiology, 41(2), 205–219. 10.1111/1.1469-8986.2004.00158.x 15032986

[psyp14110-bib-0041] Satel, S. , & Lilienfeld, S. O. (2013). Brainwashed: The seductive appeal of mindless neuroscience. Basic Civitas Books.

[psyp14110-bib-0042] Stevenson, A. (2010). Junk science (2nd ed.) Oxford Dictionary of English. https://www.oxfordreference.com/view/10.1093/acref/9780199571123.001.0001/m_en_gb0435080?rskey=ljiKbf&result

[psyp14110-bib-0043] Sutton, S. , Braren, M. , Zubin, J. , & John, E. (1965). Evoked‐potential correlates of stimulus uncertainty. Science, 150(3700), 1187–1188. 10.1126/science.150.3700.1187 5852977

[psyp14110-bib-0044] The National Registry of Exonerations . (2020). The National Registry of Exonerations. The National Registry of Exonerations. https://www.law.umich.edu/special/exoneration/Pages/about.aspx

[psyp14110-bib-0045] Verschuere, B. , Ben‐Shakhar, G. , & Meijer, E. (2011). Memory detection: Theory and application of the concealed information test. Cambridge University Press. 10.1017/CBO9780511975196

[psyp14110-bib-0046] Verschuere, B. , Crombez, G. , De Clercq, A. , & Koster, E. H. (2004). Autonomic and behavioral responding to concealed information: Differentiating orienting and defensive responses. Psychophysiology, 41(3), 461–466. 10.1111/j.1469-8986.00167.x 15102132

[psyp14110-bib-0047] Wasserman, S. , & Bockenholt, U. (1989). Bootstrapping: Applications to psychophysiology. Psychophysiology, 26(2), 208–221. 10.1111/j.1469-8986.1989.tb03159.x 2727223

