# Supplementary material for: Detection of concealed knowledge via the ERP‐based technique Brain Fingerprinting: Real‐life and real‐crime incidents
Source: Psychophysiology. 2022 Jun 7;59(11):e14110. doi: 10.1111/psyp.14110 (PMC9788041; doi:10.1111/psyp.14110)
Supplement: Supplementary file 1 — Appendix The Brain Fingerprinting Scientific Standards [file PSYP-59-e14110-s001.docx]

Detection of concealed knowledge via the ERP-based technique *Brain Fingerprinting*: Real-life and real-crime incidents

**Supplementary Material**

**Brain Fingerprinting Scientific Standards (from Farwell et al., 2013)**

The following procedures comprise the Scientific Standards for Brain Fingerprinting Tests. These Standards have been established in the peer-reviewed scientific literature, in four US patents and one UK patent, and in court documents where Brain Fingerprinting and Dr. Farwell’s testimony on it were ruled admissible as scientific evidence in court.

1. Use equipment and methods for stimulus presentation, data acquisition, and data recording that are within the standards for the field of cognitive psychophysiology and event-related brain potential research. These standards are well documented elsewhere. For example, the standard procedures Farwell introduced as evidence in the Harrington case were accepted by the court, the scientific journals, and the other expert witnesses in the case. Use a recording epoch long enough to include the full P300-MERMER. For pictorial stimuli or realistic word stimuli, use at least a 1,800-millisecond recording epoch. (Shorter epochs may be appropriate for very simple stimuli.)
2. Use correct electrode placement. The P300 and P300-MERMER are universally known to be maximal at the midline parietal scalp site, Pz in the standard International 10-20 system.
3. Apply brain fingerprinting tests only when there is sufficient information that is known only to the perpetrator and investigators. If possible, use a minimum of six probes and six targets.
4. Use stimuli that isolate the critical variable: the subject’s knowledge or lack of knowledge of the probe stimuli as significant in the context of the investigated situation. Obtain the relevant knowledge from the criminal investigator (or for laboratory studies from the knowledge-imparting procedure such as a mock crime and/or subject training session). Divide the relevant knowledge into probe stimuli and target stimuli. Probe stimuli constitute information that has not been revealed to the subject. Target stimuli contain information that has been revealed to the subject after the crime or investigated situation.
5. If initially there are fewer targets than probes, create more targets. Ideally, this is done by seeking additional known information from the criminal investigators. Note that targets may contain information that has been publicly disclosed. Alternatively, some potential probe stimuli can be used as targets by disclosing to the subject the specific items and their significance in the context of the investigated situation.
6. For each probe and each target, fabricate several stimuli of the same type that are unrelated to the investigated situation. These become the irrelevant stimuli. Use stimuli that isolate the critical variable. For irrelevant stimuli, select items that would be equally plausible for an information-absent subject. The stimulus ratio is approximately one-sixth probes, one-sixth targets, and two-thirds irrelevants.
7. Ascertain that the probes contain information that the subject has no known way of knowing, other than participation in the investigated situation. This information is provided by the criminal investigator for field studies, and results from proper information control in laboratory studies.
8. Make certain that the subject understands the significance of the probes, and ascertain that the probes constitute only information that the subject denies knowing, as follows. Describe the significance of each probe to the subject. Show him the probe and the corresponding irrelevants, without revealing which is the probe. Ask the subject if he knows (for any non-situation-related reason) which stimulus in each group is situation-relevant / crime-relevant. Describe the significance of the probes and targets that will appear in each test block immediately before the block.
9. If a subject has knowledge of any probes for a reason unrelated to the investigated situation, eliminate these from the stimulus set. This provides the subject with an opportunity to disclose any knowledge of the probes that he may have for any innocent reason previously unknown to the scientist. This will prevent any non-incriminating knowledge from being included in the test.
10. Ascertain that the subject knows the targets and their significance in the context of the investigated situation. Show him a list of the targets. Describe the significance of each target to the subject.
11. Require an overt behavioral task that requires the subject to recognize and process every stimulus, specifically including the probe stimuli, and to prove behaviorally that he has done so on every trial. Detect the resulting brain responses. Do not depend on detecting brain responses to assigned tasks that the subject can covertly avoid doing while performing the necessary overt responses.
12. Instruct the subjects to press one button in response to targets, and another button in response to all other stimuli. Do not instruct the subjects to “lie” or “tell the truth” in response to stimuli. Do not assign different behavioral responses or mental tasks for probe and irrelevant stimuli.
13. In order to obtain statistically robust results for each individual case, present a sufficient number of trials of each type to obtain adequate signal-to-noise enhancement through signal averaging. Use robust signal-processing and noise-reduction techniques, including appropriate digital filters and artifact-detection algorithms. The number of trials required will vary depending on the complexity of the stimuli, and is generally more for a field case. In their seminal study, Farwell and Donchin (1991) used 144 probe trials. In the Harrington field case, a murder case wherein brain fingerprinting and Farwell’s testimony in it were admitted in court as scientific evidence, Farwell used 288 probe trials (Farwell *et al.* 2013; Harrington v. State, 2001). In any case, use at least 100 probe trials and an equal number of targets. Present three to six unique probes in each block.
14. Use appropriate mathematical and statistical procedures to analyze the data. Do not classify the responses according to subjective judgments. Use statistical procedures properly and reasonably. At a minimum, do not determine subjects to be in a category where the statistics applied show that the determination is more likely than not to be incorrect, i.e., statistical confidence is less than 50%.
15. (a) Use a mathematical classification algorithm, such as bootstrapping on correlations, that isolates the critical variable by classifying the responses to the probe stimuli as being either more similar to the target responses or to the irrelevant responses. (b) In a forensic setting, conduct two analyses: one using only the P300 (to be more certain of meeting the standard of general acceptance in the scientific community), and one using the P300-MERMER (to provide the current state of the art).
16. Use a mathematical data-analysis algorithm that takes into account the variability across single trials, such as bootstrapping.
17. Set a specific, reasonable statistical criterion for an information-present determination and a separate, specific, reasonable statistical criterion for an information-absent determination. Classify results that do not meet either criterion as indeterminate. Recognize that an indeterminate outcome is not an error, neither a false positive nor a false negative. Error rate is the percentage of information-present or information-absent determinations that are false positives and false negatives respectively; accuracy is 100% minus the error rate.
18. Restrict scientific conclusions to a determination as to whether or not a subject has the specific situation-relevant knowledge embodied in the probes stored in his brain. Recognize that brain fingerprinting detects only presence or absence of information – not guilt, honesty, lying, deception, or any action or non-action. Do not offer scientific opinions on whether the subject is lying or whether he committed a crime or other act. Recognize that the question of guilt or innocence is a legal determination to be made by a judge and jury, not a scientific determination to be made by a scientist or a computer.
19. Evaluate error rate / accuracy based on actual ground truth. Ground truth is the true state of what a scientific test seeks to detect. Brain fingerprinting is a method to detect information stored in a subject’s brain. Ground truth is whether the specific information tested is in fact stored in the subject’s brain. Establish ground truth with certainty through post-test interviews in laboratory experiments and in field experiments wherein subjects are cooperative. Establish ground truth insofar as possible through secondary means in real-life forensic applications with uncooperative subjects. Recognize that ground truth is the true state of what the subject in fact knows, not what the experimenter thinks the subject should know, not what the subject has done or not done, and not whether the subject is guilty, or deceptive.
20. Make scientific determinations based on brain responses. Do not attempt to make scientific determinations based on overt behavior that can be manipulated, such as reaction time.
